# Cell–Cell Communication Alterations *via* Intercellular Signaling Pathways in Substantia Nigra of Parkinson’s Disease

**DOI:** 10.3389/fnagi.2022.828457

**Published:** 2022-02-25

**Authors:** Maoxin Huang, Liang Xu, Jin Liu, Pei Huang, Yuyan Tan, Shengdi Chen

**Affiliations:** ^1^Department of Neurology and Institute of Neurology, Ruijin Hospital, Shanghai Jiao Tong University School of Medicine, Shanghai, China; ^2^Research Center for Translational Medicine, East Hospital, Tongji University School of Medicine, Shanghai, China; ^3^Lab for Translational Research of Neurodegenerative Diseases, Shanghai Institute for Advanced Immunochemical Studies, Shanghai Tech University, Shanghai, China

**Keywords:** Parkinson’s disease, sc/snRNA-seq, CellChat, dopaminergic neurons, cell–cell communication, signaling

## Abstract

Parkinson’s disease (PD) is a neurodegenerative movement disorder characterized with dopaminergic neuron (DaN) loss within the substantia nigra (SN). Despite bulk studies focusing on intracellular mechanisms of PD inside DaNs, few studies have explored the pathogeneses outside DaNs, or between DaNs and other cells. Here, we set out to probe the implication of intercellular communication involving DaNs in the pathogeneses of PD at a systemic level with bioinformatics methods. We harvested three online published single-cell/single-nucleus transcriptomic sequencing (sc/snRNA-seq) datasets of human SN (GSE126838, GSE140231, and GSE157783) from the Gene Expression Omnibus (GEO) database, and integrated them with one of the latest integration algorithms called Harmony. We then applied CellChat, the latest cell–cell communication analytic algorithm, to our integrated dataset. We first found that the overall communication quantity was decreased while the overall communication strength was enhanced in PD sample compared with control sample. We then focused on the intercellular communication where DaNs are involved, and found that the communications between DaNs and other cell types via certain signaling pathways were selectively altered in PD, including some growth factors, neurotrophic factors, chemokines, etc. pathways. Our bioinformatics analysis showed that the alteration in intercellular communications involving DaNs might be a previously underestimated aspect of PD pathogeneses with novel translational potential.

## Introduction

Parkinson’s disease (PD) is the second most common neurodegenerative disease (ND) worldwide (Ascherio and Schwarzschild, 2016). The heterogeneous etiology of PD is not fully understood, and the interplay between aging, environmental and genetics factors was proposed to be responsible (Pang et al., 2019). The pathology of PD is characterized with the intracellular α-synuclein-containing inclusion called Lewy body and the progressive loss of dopaminergic neurons (DaNs) in the substantia nigra (SN) (Dinter et al., 2020). Therefore, the spotlight of PD research has justifiably been casted on the SN DaN themselves. In the course of PD, the primary victims DaNs suffer multiple intracellular dysfunctions and deregulation, including but not limited to mitochondria dysfunction, oxidative stress, autophagic dysfunction, α-synuclein aggregation and so on (reviewed in Subramaniam and Chesselet, 2013; Xilouri et al., 2016; Hou et al., 2020). Despite a considerable leap forward in decoding these mechanisms, they did not yield much in translatable diagnostic and therapeutic strategies, leaving a giant gap between basic science and clinical application.

However, no paradox, no progress. This gap also tells us that the selective susceptibility of DaNs in PD is not completely intrinsic to DaNs themselves, and those pathologic alterations inside DaN may not constitute the whole story of PD pathogeneses. Now, emerging clues reveal that the rest of this “story” may lie outside of DaNs and possibly between DaNs and other cells. Indeed, normal brain functions necessitate intact integrity, where different neurons and non-neuronal cells interact with each other in an orchestrated and tightly regulated manner. So far, multiple intercellular communication in the central nervous system (CNS) have been characterized, including but not limited to neuron/microglia (reviewed in Posfai et al., 2019; Cserep et al., 2021), neuron/astrocyte (Salmina, 2009; Durkee and Araque, 2019; Khakh, 2019), neuron/oligodendrocyte (ODC) (Gibson et al., 2014; Mitew et al., 2018; Stedehouder et al., 2018; Jang et al., 2019; Ortiz et al., 2019; Xin et al., 2019), neuron/oligodendrocyte precursor cell (OPC) (Hardy and Reynolds, 1993; Bergles et al., 2000; Lin and Bergles, 2004; Maldonado and Angulo, 2015; Orduz et al., 2015; Venkatesh et al., 2015; Voronova et al., 2017; reviewed in Watson et al., 2020), neuron/endothelial (Guo et al., 2008), endothelial/OPC (Arai and Lo, 2009), and microglia/astrocyte (reviewed in Jha et al., 2019) communications. These communications are mainly mediated through intercellular signaling molecules, including growth or differentiation factors, neurotrophic factors, cytokines, kinins, etc., which are busy messengers relaying signals and are essential to neuronal functions and survival. These intricate cell–cell communication networks are somehow perturbed in NDs, such as Alzheimer’s disease (AD), where the homeostatic communications among neurons, glial cells, and vascular cells are altered, resulting in pathologic events like neuroinflammation. For example, the neurovascular unit, which contains nearly every cell type in the brain and constitutes a dynamic interactive web, is severely disturbed in AD, which hold great potential for therapeutic intervention (reviewed in Salmina, 2009; Henstridge et al., 2019). However, similar perspective has seldom been acknowledged in other NDs. In PD, some scattered evidences have partly depicted cell-cell communication involving DaNs. For example, glial cells secrete neurotrophic factors essential for DaN (reviewed in Kramer and Liss, 2015; Lindholm et al., 2016; Palasz et al., 2020). Reactive astrocytes and microglia interact with and affect DaN in neuroinflammation under PD condition (reviewed in Kwon and Koh, 2020; Pajares et al., 2020). However, these signaling molecules are seldom coupled with their senders and receivers to construct a complete cell–cell communication pathway. In addition, the existing knowledge of intercellular signaling pathway-mediated communication are incomplete and fragmented, lacking a systems-level analysis and a global picture. In the meantime, the effects of these cell–cell interactions on the function and survival, as well as the dysfunction and loss, of DaN, are still unknown.

To further explore the roles of intercellular communication in the dysfunction and degeneration of DaNs at a systemic level, we harvested three online published single-cell/single-nucleus transcriptomic sequencing (sc/snRNA-seq) datasets of human SN (GSE126838, GSE140231, and GSE157783) from the Gene Expression Omnibus (GEO) database, and integrated them with a recently developed integration algorithm called Harmony. At this step, we found that SN’s cell composition was altered in PD, especially for some neuronal populations. We then analyzed the intercellular communication in both healthy donors and PD patients with CellChat, the latest algorithm that can infer intercellular communication with sc/snRNA-seq data (Jin et al., 2021). We found a decrease in global communication quantity and an increase in overall communication strength in PD sample compared with healthy sample. By dissecting the global communications, we found that several intercellular signaling pathways are altered in PD, and those involving DaN include some growth factors, neurotrophic factors, neuropeptides, etc. pathways. Our data showed widespread alterations in cell–cell communications in PD, implicating its importance in the PD mechanisms.

## Results

### Integration of Online Published sc/scRNA-seq Datasets of Human Substantia Nigra

**FIGURE 1:**
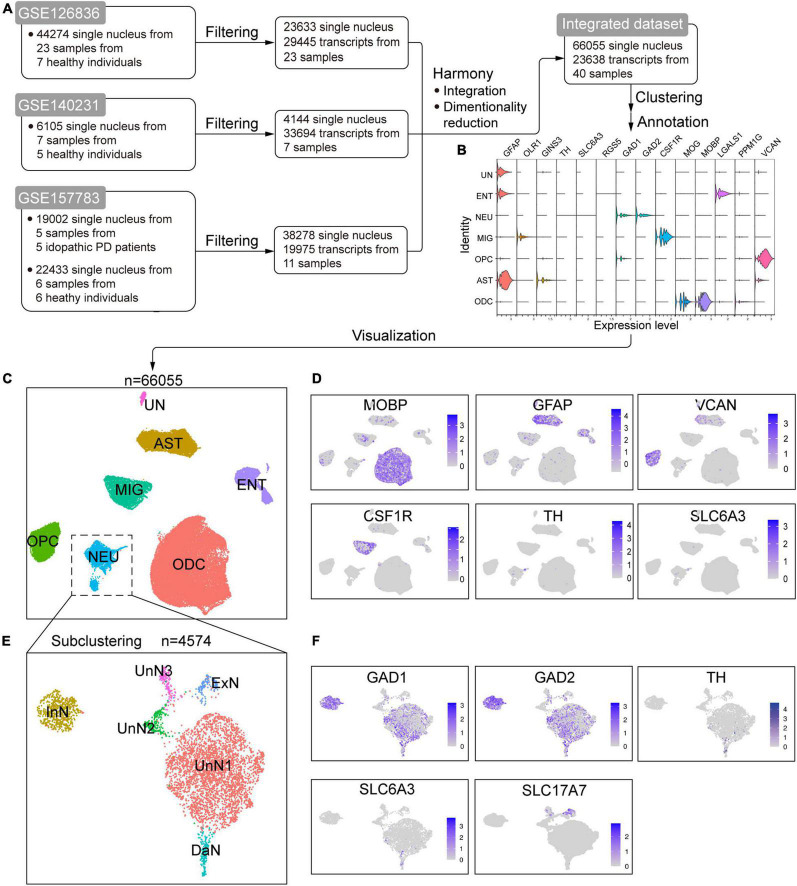
Integration of online published single-cell/single-nucleus transcriptomic sequencing (sc/snRNA-seq) datasets of postmortem human substantia nigra (SN). **(A)** Schematic flowchart showing the processing, integration, and generation of an integrated dataset from online published sc/snRNA-seq datasets of human SN. **(B)** Violin plot of the expression of canonical marker genes in the identified cell populations. **(C)** Uniform manifold approximation and projection (UMAP) plot of the identified cell populations. **(D)** UMAP feature plots of the expression of some well-known marker genes in the cell clusters. **(E)** UMAP plot of the identified neuron subtypes after subclustering the NEU cluster in C. **(F)** UMAP feature plots of the expression of well-known marker genes in the identified neuron subpopulations. ODC, oligodendrocyte cluster; AST, astrocyte cluster; OPC, oligodendrocyte precursor cell cluster; MIG, microglia cluster; NEU, neuron cluster; ENT, endothelial cell cluster; UN, unidentified cell cluster; InN, inhibitory neuron cluster; ExN, excitatory neuron cluster; DaN, dopaminergic neuron cluster; UnN1, unidentified neuron cluster 1; UnN2, unidentified neuron cluster 2; UnN3, unidentified neuron cluster 3.

To study the alteration of cellular composition associated with PD, we merged three recently published sc/scRNA-seq datasets of post mortem human SN (GSE126836, GSE140231, and GSE157783) (Welch et al., 2019; Agarwal et al., 2020) to yield a new combined single-cell dataset (Figure 1A and Supplementary Figure 2). The basic information of the three individual datasets and our integrated dataset were shown in Figure 1A, Supplementary Figure 3, and Supplementary Tables 1, 2. After clustering and visualization (see section Materials and Methods), cells were grouped into seven clusters. We manually annotated these clusters with marker genes or identified highly expressed genes (Figures 1B–D and Supplementary Figure 5). The marker genes were well-acknowledged based on the original sc/snRNA-seq study (Welch et al., 2019; Agarwal et al., 2020; Smajić et al., 2020). We also performed differential gene analysis (Supplementary Table 3) and Gene Ontology (GO) and Kyoto Encyclopedia of Genes and Genomes (KEGG) enrichment analysis (Supplementary Figures 6, 7) for each cluster to supplement the cell identity annotation. The oligodendrocyte cluster (ODC) is marked by dense expression *MOBP* and *MOG*, with two ODC subtype annotated by *LGALS1* and *PPM1G*, respectively. ODC is enriched in genes associated with myelin and myelination, ensheathment of neuronal axon, axonogenesis, etc., well supporting its identity. The astrocyte cluster (AST) is characterized with *GFAP* and some *GINS3* expression. AST is enriched in genes associated with extracellular structural organization, axonogenesis, regulation of neuron and neuronal projection development, synapse, cell adherence, calcium signaling, etc., in line with the roles of astrocytes as a structural glue, in neuronal development, in brain homeostasis of internal environment and transmitter, in synaptic function, and calcium-mediated excitability (reviewed in Sofroniew and Vinters, 2010). The oligodendrocyte precursor cell cluster (OPC) is consistently marked by *VCAN*, and the differently expressed genes are enriched in synaptic related structures and functions, glutamatergic synapse and receptors, and calcium signaling pathways. The microglia cluster (MIG) is annotated with *OLR1* and *CSF1R*, and its genes are enriched in immune response and immune cell activation, phagocytosis, endocytosis, viral and bacterial infection, chemokine signaling, etc. The neuron cluster (NEU) is characterized with uneven expression of *GAD1*, *GAD2*, and scarce expression of *TH* and *SLC6A3*, hinting at glutaminergic neurons and DaN, respectively. Genes in NEU are enriched in neuronal features, including synaptic structures and activities, ion channel and neurotransmission. The remaining two clusters are hard to annotate because of the lack of canonical marker genes expression. However, through GO/KEGG analysis, we found that one of them was enriched in genes associated with endothelium (endothelial migration, differentiation, development, etc.), vasculature, cell junctions, adhesions, etc. Although this cluster showed little expression of endothelial marker *RGS5* (Agarwal et al., 2020), we annotated it as endothelial cells (named ENT) based on GO/KEGG analysis results. The last cluster cannot be annotated by well-known marker genes as well, but genes in this cluster are enriched in cilium-related structures and activities, such as cilium organization, assembly and movement. However, there has been so far no cell identity in the SN corresponding to these functions, and we ultimately labeled this cluster as “unidentified” cluster (UN).

We noticed that NEU is a mixed cluster comprising different types of neurons. We thus subclustered NEU to identify and annotate them with known marker genes and differentially expressed genes (Figures 1E,F and Supplementary Table 4). We identified an inhibitory neuron cluster (named InN) marked by *GAD1* and *GAD2*, a dopaminergic neuron cluster (DaN) characterized by *TH*, *SLC6A3*, *ALDH1A1*, and *SLC18A2*, and an excitatory neuron cluster (named ExN) marked by *SLC17A7*. The remaining three neuronal subclusters cannot be annotated by canonical marker genes. One of them (referred to as unidentified neuron cluster 1, UnN1 hereafter) highly expresses *KLHL1*, and about half cells expresses *GAD1* and *GAD2*. Another (referred to as UnN2) highly expressed *PTPN3*, *CASQ2*, *GRID2IP*, etc., and the third (referred to as UnN3) highly expresses *FAT2*, *CRTAM*, *LINC01798*, etc.

### Integrated sc/snRNA-seq Data Show Parkinson’s Disease-Associated Alterations in Cell Composition

**FIGURE 2:**
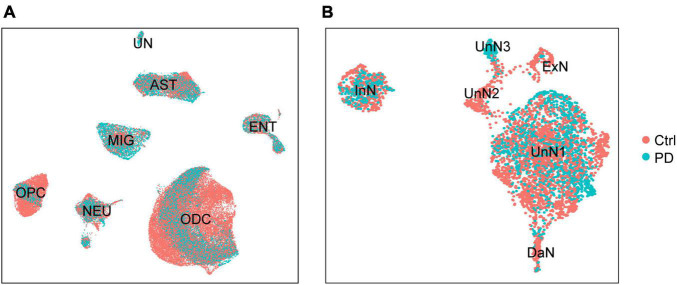
The integrated dataset reveals PD-associated cell composition alteration in PD. **(A)** UMAP plot of the distribution of control (Ctrl) and PD donors-derived cells. **(B)** UMAP plot of the distribution of control and PD donors-derived neurons.

Loss of DaN in the SN are the pathologic hallmark for PD. In addition to DaN loss, overall cell composition is also altered in PD SN. One individual sc/snRNA-seq data (Smajić et al., 2020) showed that the number of microglia and astrocytes were increased, whereas that of ODCs was decreased in the midbrain of PD patients compared with healthy individuals. Importantly, a PD-specific neurons cluster highly expressing *CAPDS2* was found, purported as a group of DaN losing normal identity and functions (Smajić et al., 2020). To further explore cell composition alterations with the integrated data, we distinguished single cells by their origin of either control or PD sample (Figure 2). Across the global clustering, single cells from both control and PD sample coexist in each cluster, supporting a successful removal of batch effect and a lack of alteration in overall cell composition. The composition across different cell clusters is not greatly altered, whereas the composition within each cell cluster is slightly altered in PD sample compared with control sample (Figure 2A). Further, when we zoomed in on the NEU, we found great differences in neuronal composition between control and PD sample (Figure 2B). Strikingly, UnN3 is almost exclusively composed by cells from PD sample. UnN3 is characterized by high expression of genes associated with vesicle trafficking (such as *EXPH5* and *CADPS2*), brain development (such as *PAX6*, *MEIS1*, *CHD7*, *CECR2*, *DPF3*), transcription regulation (*PAX6*, *MEIS1*), chromatin remodeling (such as *CECR2*, *CHD7*, *DPF3*), and cell proliferation and differentiation (such as *FGF5*) (Supplementary Table 4). GO/KEGG enrichment analysis showed that UnN3 was enriched in genes associated with calcium and cAMP signaling pathways (Supplementary Figure 8).

Dopaminergic neuron loss and neuroinflammation are well-established pathologic features of PD. We set out to explore whether our integrated data reflects these hallmarks of PD. We found that microglia and astrocyte population were significantly increased in PD sample compared with control sample (Supplementary Figures 4B,C; Mann–Whitney test, *p* = 0.0002 for microglia, *p* = 0.0020 for astrocyte). However, there was no obvious dopaminergic loss in PD sample (Supplementary Figure 4A; Mann–Whitney test, *p* = 0.1796), in line with Smajić’s study where the PD sample-containing dataset GSE157783 was created (Smajić et al., 2020). The absence of dopaminergic neuron loss in their and our integrated dataset was, at least partially, because a great number of nucleus of dopaminergic neurons (10-20 μm in diameter) were lost in the 15 μm-thick tissue sectioning procedure (Smajić et al., 2020).

### Integrated sc/snRNA-seq Data Show Alterations in Cell–Cell Communications in Parkinson’s Disease

As previously mentioned, there is a vacancy of an overview of cell–cell communications in either healthy or PD SN, especially those involving DaN. Because of many-to-many patterns of molecule-to-cell and sender-to-recipient relations, it is impractical to systemically study the full picture of cell–cell communication with manual labor. Therefore, we applied a recently established algorithm called CellChat to study intercellular signaling communications based on gene expression information derived from single-cell sequencing data (Jin et al., 2021). CellChat enables robust quantitative inference, analysis and visualization of intercellular communication network based on known knowledge of ligand/receptor pairs and other signaling cofactors (Jin et al., 2021). To be focused and relevant to the PD pathology, we primarily characterized the communication networks that involve DaN, with only a quick glimpse of those that do not involve them. We compared the communication patterns between the control and PD sample to reflect or predict PD-associated pathologic alterations in cell–cell communication.

#### Global Alterations of Intercellular Signaling Network in Parkinson’s Disease

**FIGURE 3:**
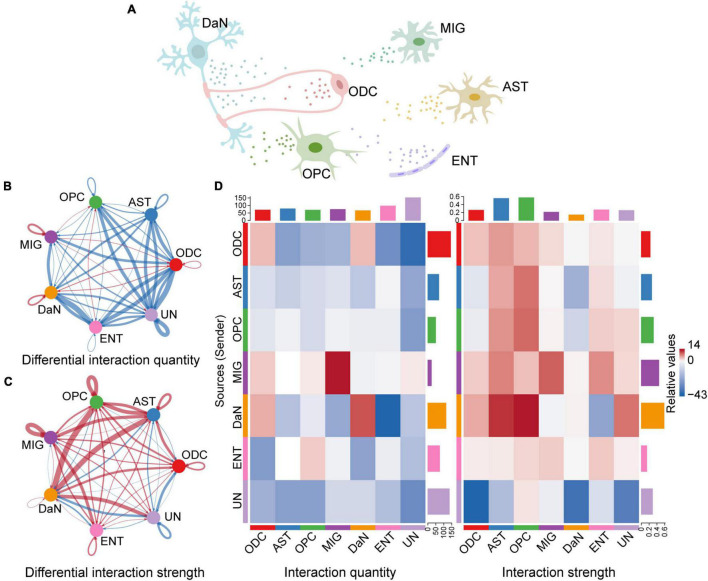
Inference of cell–cell communications by CellChat shows global alterations in signaling pathways-mediated communications between DaN and non-neuronal cells in PD. **(A)** Schematic diagram of cell–cell communication between DaN and non-neuronal cells. Circle plots of the interaction quantity **(B)** and interaction strength **(C)** between DaN and non-neuronal cells. Blue lines indicate that the displayed communication is decreased in PD, whereas red lines indicate that the displayed communication is increased in PD compared with healthy control. **(D)** Heatmaps of the interaction quantity (left panel) and interaction strength (right panel) between DaN and non-neuronal cells. Blue color indicates that the displayed communication is decreased in PD, while red color indicates that the displayed communication is increased in PD compared with healthy control.

We first quantified and visualized the global communication atlas between DaN and other neuronal populations by CellChat, and compared the data between the control and PD sample. We found that the inferred number of interactions among neurons is generally decreased in PD compared with that under physiological condition. By contrast, the interaction strength among neurons is overall enhanced in PD (Supplementary Figure 9). We then studied the overall interactions between neurons and non-neuronal cells (Figure 3A) in a similar way with CellChat. However, as NEU is a mixture of different types of neurons, it would be confusing to study the communications involving multiple neuronal types simultaneously. To be more specific and relevant, we chose to focus on DaN, and thus removed other types of neurons in the NEU. Therefore, the original NEU turned into a cluster only containing DaN. Our following analysis involving non-neuronal cells were all based on this modification made to the integrated dataset. This process enabled us to study the communications between DaN and the previously annotated non-neuronal cells. We found that the overall interaction number between DaN and other non-neuronal cell clusters also decreases in PD compared with that under physiological condition. By contrast, the interaction strength increases in PD (Figures 3B–D). Taken together, the global communication involving DaN decreases in quantity while increases in strength in PD.

#### Alterations of Individual Signaling Pathways in Parkinson’s Disease

**FIGURE 4:**
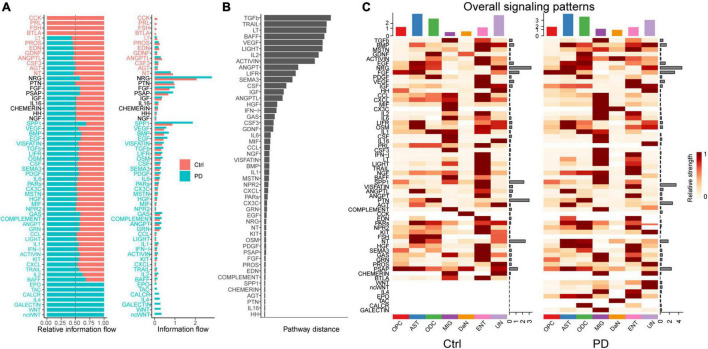
Cell–cell communications mediated by individual signaling pathways are altered in PD between DaN and non-neuronal cells. **(A)** Bar plots of the ranking of signaling axes by overall information flow differences in the interaction networks between control (Ctrl) and PD sample. The top signaling pathways with red-colored labels are more enriched in the control sample, the middle ones with black-colored labels are equally enriched in control and PD sample, and the bottom ones with green-colored labels are more enriched in the PD sample. **(B)** Bar plot of the ranking of signaling axes between control and PD sample by pairwise Euclidean distance. **(C)** Heatmaps of the overall (comprising both outgoing and incoming) signaling flows of each cell population mediated by individual signaling axes in control and PD sample.

To dissect this global alteration, we first calculated the information flows for each signaling pathway, which is defined as total communication probability among all the pairs of cell groups in the communication network (Jin et al., 2021). Among neurons (Supplementary Figure 10A), the CRH, PACAP, PROS, and some other signaling communication pathways are turned off, whereas the CCL, VEGF, GDF, and VISFATIN are turned on in PD compared with control. Some signaling pathways like GDNF, NGF and PARs are decreased, whereas some others like ANGPT, EGF, and HGF are increased in PD. Some pathways like NPR2 and BMP are comparable between control and PD. Between DaN and other non-neuronal cells (Figure 4A), CCK, PRL, FSH, and BTLA signaling communication pathways are turned off while the WNT, galectin, IL-4, CALCR, TAC and EPO pathways are turned on in PD. Some pathways (like GDNF) are decreased and some increased (like SPP1, EGF, TFGb, CX3C) in PD compared with control, whereas some pathways (including NRG, PTN, FGF, PSAP) are comparable between control and PD.

We then used another method to dissect the overall alteration by computing the Euclidean distance to measure the dissimilarity between any pair of the shared signaling pathways. Some signaling pathways, such as NRG, PRL, NGF, etc. among neurons (Supplementary Figure 10B), and TGFb, TRAIL, LT, etc. between DaN and non-neuronal cells (Figure 4B), have a larger Euclidean distance than other signaling pathways, implying that these pathways are greatly altered in PD compared with control.

Besides, we also characterized communication patterns with pattern recognition methods. The results shown as riverplots demonstrated that the patterns of communication among different neuronal populations (Supplementary Figure 11) and between DaN and non-neuronal cells (Supplementary Figure 12) differed greatly between control and PD. These differences reveal the alteration of the functional cooperation between cells via signaling pathways in PD.

#### Alterations of Signaling Pathways Involving Dopaminergic Neuron in Parkinson’s Disease

We have shown that intercellular signaling networks are altered on a global scale in PD. We have also shown that the changes of individual signaling pathways underlies this global alteration without discriminating the interacting cell clusters. To focus on alterations most likely relevant to PD, we distinguished the signaling pathways that involves DaN and that are greatly altered in PD (Figure 4C and Supplementary Figures 10C, 13). These signaling pathways include but not limit to some neurotrophic factors, growth and/or differentiation factors, neuropeptides and chemokines, which will be detailed in the following paragraphs.

**FIGURE 5:**
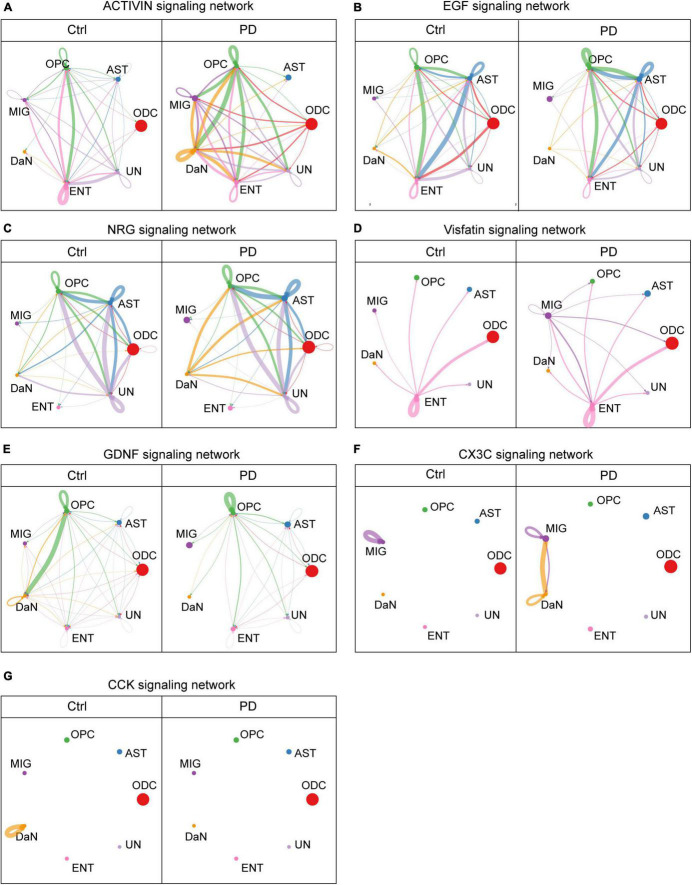
Some cell–cell communications between DaN and non-neuronal cells mediated by signaling pathways are greatly altered in PD compared with control (Ctrl) sample. Circle plots show and compare cell–cell communication alterations between DaN and non-neuronal cells mediated by some of the signaling axes, including activins **(A)**, epithelial growth factor (EGF) **(B)**, neuregulins (NRGs) **(C)**, Visfatin **(D)**, glial cell line-derived neurotrophic factors (GDNF) **(E)**, CX3C **(F)**, and cholecystokinin (CCK) **(G)**.

Growth and/or differentiation factors promote cell development. Among them, we found that intercellular activin signaling among different types of neurons (Supplementary Figure 14A) are overall decreased in PD. The hub of activin signal is normally located at excitatory neurons (ExN), which is also lost in PD. Between DaN and non-neuronal cells (Figure 5A), the activin signals, on the contrary, are generally increased in PD compared with control. The hub of activin signal is normally located at endothelial cells, and this endothelial cells-centered pattern is lost due to the increasing involvements of OPCs, microglia and DaN in activin signaling network in PD. For DaN, they are marginally involved in the intercellular activin network under physiological condition. However, DaN is intensively involved as a signaling center of activin in their interaction with non-neuronal cells in PD.

Besides great alteration in activin signals, we also found another growth factor signals, the epithelial growth factor (EGF), manifests a totally different alteration pattern. Under physiological condition, the EGF signals among neurons (Supplementary Figure 14B) are only composed of a bidirectional communication between the DaN and the UnN1 neurons, as well as an autocrine signaling pattern of the UnN1 neurons. DaN also send weak outgoing EGF signals to some non-neuronal cells (Figure 5B). This leading bidirectional signal between DaN and UnN1 neurons are completely lost in PD, whereas the overall abundance of EGF signals increases in PD, with more involvement of other types of neurons. In addition, the outgoing signals from DaN to non-neuronal cells are slightly weakened without obvious perturbation in PD.

We also discovered neuregulins (NRGs), whose signals are also greatly altered in PD. NRG signals are abundant among astrocytes, OPCs and the UN cluster in both conditions (Figure 5C). Under physiological condition, DaN release no NRG signals to other neurons (Supplementary Figure 14C), and release weak NRG signals to ODCs, astrocytes, OPCs and the unidentified clusters. But in PD, DaN manifest strengthened outgoing signals to other neurons (UnN2 and UnN3) and the abovementioned non-neuronal cells.

Interestingly, we found nicotinamide phosphoribosyltransferase (NAMPT, also known as Visfatin), which is both an intracellular enzyme that catalyzes NAD^+^ biosynthesis and an extracellular secreted factors with growth factor, enzymatic and cytokine activities (reviewed in Carbone et al., 2017). Under physiological condition there is no Visfatin signals among neurons (Supplementary Figure 14D), and the visfatin signal between DaN and non-neuronal cells (Figure 5D) only involves endothelial cells. But the UnN2 neurons begin to release strong Visfatin signals to other neurons in PD, including UnN2 neurons themselves, and microglia begin to release Visfatin signals to all other non-neuronal cells and DaN.

Another group of intercellular signaling molecules are neurotrophic factors. The glial cell-derived neurotrophic factors (GDNFs) are potent neurotrophic factor for the midbrain DaN (Lin et al., 1993). Among neurons (Supplementary Figure 14E), the normal GDNF signals among UnN1, UnN2 and DaN are abolished, whereas an intense autocrine GDNF signaling pattern of UnN1 emerge in PD. Among DaN and non-neuronal cells (Figure 5E), OPCs are the main source of GDNF ligand, and DaN are the main receivers of the GDNF signaling. The GDNF signaling network involving DaN are globally attenuated in PD. More specifically, the GDNF signal from OPCs to DaN are severely dampened, and those from ODCs, astrocytes, microglia, endothelial cells and unknown cells to DaN are completely lost in PD. In another direction, the outgoing GDNF signal from DaN to non-neuronal cells are also weakened, and the autocrine GDNF signal in the DaN is also abolished in PD.

Chemokines are signaling molecules that can induce leukocyte migration and trafficking. CX3C is a branch of chemokines that only contains one single ligand CX3CL1 and one receptor CX3CR1. We found that there was no observable interaction of CX3C signal among different cells, except an autocrine signal of microglia under physiological condition. However, there emerge intense bidirectional interactions of CX3C signal between DaN and microglia in PD (Figure 5F and Supplementary Figure 14F). Meanwhile, the autocrine CX3C signal of DaN emerge, whereas that of microglia deteriorates. In other words, DaN form crosstalk with microglia via CX3CL1-CX3CR1 signaling in PD.

Neuropeptides are intercellular messengers released by neurons that can either modulate neurotransmitters or act as hormones. Cholecystokinin (CCK) is both a gastrointestinal peptide and a neuropeptide. We found that intercellular CCK signal is scarce under physiological condition, with a single autocrine CCK signal mediated by CCK-CCK_B_ receptor in DaN. In PD, the autocrine CCK signal of DaN disappears, and an autocrine CCK signal of the excitatory neurons (ExN) emerges (Figure 5G and Supplementary Figure 14G).

## Discussion

The emerging application of scRNA-seq has spurred a considerable leap in life science. Through unbiased transcriptomic characterization of individual cells within a given population, scRNA-seq discloses uniqueness of each individual cells, reveals cellular heterogeneity and tissue composition, facilitates discovery of novel cell populations, monitors transcription dynamics and models cellular developmental trajectory, and unveils mechanisms of gene expression regulation (reviewed in Kolodziejczyk et al., 2015; Hedlund and Deng, 2018; Luecken and Theis, 2019). These enable *de novo* biological discovery that are unimaginable in bulk analysis. scRNA-seq techniques also pushes the boundaries of PD research. Several research groups have performed scRNA-seq on the human (La Manno et al., 2016; Welch et al., 2019; Agarwal et al., 2020) or mouse midbrain or SN (La Manno et al., 2016; Hook et al., 2018). Thanks to scRNA-seq, the genetic risks of PD were found associated with different cell-type-specific gene expression patterns within the midbrain or SN (Agarwal et al., 2020; Bryois et al., 2020; Smajić et al., 2020), highlighting the contribution of different cell types in PD etiology. The population-specific data of mouse DaN harvested from scRNA-seq were utilized to establish a gene scoring system for prioritizing candidate genes in the GWAS intervals implicated in the risk of PD (Hook et al., 2018). The upregulated or downregulated genes in PD were specifically expressed by some cell types (Bryois et al., 2020). In addition, the cell composition of midbrain was found altered in PD patients with the alteration in glial numbers and profiles and the emergence of abnormal DaN (Smajić et al., 2020). scRNA-seq data also showed the diversity midbrain DaN during development (La Manno et al., 2016; Kee et al., 2017; Tiklova et al., 2019).

### Integrated sc/snRNA-seq Dataset Offers Insights Into Parkinson’s Disease-Associated Cellular Alterations

In our study, we integrated three scRNA-seq datasets of human SN published online. Although scRNA-seq data from one dataset might be sufficient to offer some novel insights, joint and integrated analyses of datasets of different origins have extra advantages, as it boosts the statistical power and facilitates the identification of rare cell populations that are impossible to be discovered in the original individual dataset (Butler et al., 2018; Korsunsky et al., 2019). Moreover, studying the disease-specific cell alteration also necessitates data integration. Proper data integration can preserve valuable biological variance meanwhile removing batch effects. Several multi-dataset integration algorithms were developed, and we used the latest algorithm called Harmony, which solves several key challenges of joint embedding of scRNA-seq data (Korsunsky et al., 2019), for integration.

By characterizing cell composition in the SN, we found PD-related cell population alterations. We found increases in microglia and astrocyte population per sample in PD sample compared with control sample (Supplementary Figures 4B,C), reflecting a neuroinflammatory condition in PD. However, we failed to observe the loss of dopaminergic neurons in our integrated analysis, which is in line with the Smajić’s study (Smajić et al., 2020). In their study, Smajić et al. found no significant difference of dopaminergic neuron population between PD and control samples, and dopaminergic neurons accounted for too small a portion (0.18%) of total cells in their snRNA-seq data. They concluded that it was because the 15μm sectioning thickness they chose was smaller than the 10−20 μm diameter of the dopaminergic neuron nucleus, so that they “lost” a great number of intact nucleus, preventing a reliable comparison between PD and control sample. They further confirmed that dopaminergic neuron loss did exist in their PD samples, as immunofluorescence for neuromelanin (NM), tyrosine hydrolase (TH) and MAP2 confirmed a significant decrease of dopaminergic neurons in PD samples.

An interesting finding was that the neuronal subcluster UnN3 was almost exclusively composed of PD donor-derived cells, with rare healthy donors-derived cells. This PD-specific cluster comprise neurons that are almost exclusively from PD sample and scarcely from control sample, implying its close implication in PD. Smajić et al. (2020) also discovered a neuronal cluster exclusively present in PD samples, which overexpressed *CAPDS2* and *TIAM1*. These neurons were largely positive for neuromelanin (NM) but negative for tyrosine hydrolase (TH). In addition, they showed that the *CAPDS2-*overexpressed neurons transcriptionally deviated from a differentiated signature of mature neurons toward an undifferentiated signature of neuroblasts, and a considerable portion of them entered the cell cycle. They concluded that these cells were the injured dopaminergic neurons, which lost the original identity and attempted to, though probably unsuccessfully, restart the proliferative or developmental program (Smajić et al., 2020).

Interestingly, the UnN3 in our joint analysis also highly expressed *CADPS2* and *TIAM1*. It was characterized by high expression of genes functioning in vesicle trafficking (such as *EXPH5* and *CADPS2*), brain development (such as *PAX6*, *MEIS1*, *CHD7*, *CECR2*, *DPF3*), transcription regulation (*PAX6*, *MEIS1*), chromatin remodeling (such as *CECR2*, *CHD7*, *DPF3*), and cell proliferation and differentiation (such as *FGF5*) (Supplementary Table 4). Because the transcriptional signature was similar between Smajić’s *CAPDS2-*overexpressed cluster and our UnN3 cluster, we inferred that our UnN3 cluster might correspond to their *CADPS2*-overexpressed neurons.

In addition to evidences in favor of their homogeneity, our joint analysis further yielded some detailed and novel information about this population. For example, GO/KEGG enrichment analysis showed that UnN3 highly expressed genes related to calcium and cAMP signaling pathways (Supplementary Figure 8 and Supplementary Table 4). Looking closer to the top 20 differentially expressed genes (DEGs), we found that UnN3 is enriched in highly expressed genes associated with brain development. Among these genes, *CHD7* encodes a chromatin remodeler protein called chromodomain-helicase-DNA-binding protein 7 (CHD7), which controls neural differentiation via transcriptional regulation (reviewed in Feng et al., 2017). Another brain development-related DEG, *PAX6*, encodes a transcription factor (PAX6) involved in the differentiation and proliferation of neural stem cells and progenitor cells, as well as the specification, migration and maintenance of neurons (reviewed in Kikkawa et al., 2019). PAX6 is also involved in the guiding of dopaminergic neuronal projection from the SN to ventral tegmental area (VTA) during development (Vitalis et al., 2000). Importantly, PAX6 has been implicated in PD: human postmortem study showed that *PAX6* was expressed in a small portion of cells in the SN, and animal study showed that some of the PAX6-positive cells in the SN are differentiated dopaminergic neurons (Thomas et al., 2016). In addition, they reported a significant loss of PAX6-positive cells in postmortem SN tissues of advanced PD patients, and an early significant increase in PAX6-positive cells in 6-OHDA lesioned PD rat model. However, they did not characterize the expression level of *PAX6*, and our snRNA-seq data showed that PAX6 is significantly upregulated in a neuronal subpopulation almost exclusive to PD sample. In addition, Thomas et al. (2016) reported a pro-survival role of PAX6 in PD experimental models. Another brain development-related DEG, *MEIS1*, encodes the transcription factor homeobox protein Meis1 that functions in controlling the state of stem or progenitor cells (Heine et al., 2008; Tucker et al., 2010). Interestingly, Meis1 and PAX6 are closely related, because Meis1 can activate the transcription of *PAX6* by binding to the enhancer of *PAX6* (Owa et al., 2018). Another development-related DEG, *TIAM-1*, encodes a GDP-GTP exchange factor (GEF) for Rho-like GTPase Rac-1 (Michiels et al., 1995). TIAM-1 is also involved in brain development (Yoo et al., 2012), where it regulates cell migration and neurite outgrowth (Ehler et al., 1997; Demarco et al., 2012). More specifically, TIAM-1 is expressed in the ventral midbrain and necessary for the differentiation of midbrain dopaminergic neurons (Cajanek et al., 2013). Another highly expressed gene in UnN3, *GRIN2C*, encodes the 2C subunit of the *N*-methyl-D-aspartate (NMDA) receptor (GluN2C). Previous studies have reported the expression of *GRIN2C* in midbrain (Allgaier et al., 1999) and SN (Ravikrishnan et al., 2018). Among NMDA receptor subunits, GluN2C is characterized with low sensitivity to magnesium block, low conductance, low calcium permeability and low opening probability. It was reported that neuronal GluN2C confer protective effects, whereas oligodendrocytic and astrocytic GluN2C mediates detrimental effects (reviewed in Ding et al., 2020). Therefore, the high expression of *GRIN2C* in the neuronal subcluster UnN3 might mediate protective effects. Interestingly, the expression of *TIAM-1* and *GRIN2C* are both synergistically regulated by the expression and phosphorylation of the transcription factor ETV1, whose encoding genes *ETV1* is another highly expressed genes within the top five DEGs in UnN3. Therefore, the three highly expressed genes in UnN3, *GRIN2C*, *TIAM-1*, and *ETV1* are closely related. Considering the co-upregulation of two groups of associated genes, namely *PAX6*/*MEIS1* and *TIAM-1*/*GRIN2C*/*ETV1*, as well as other developmental genes, it is likely that the UnN3 neurons might attempt to initiate multiple responsive machineries that involves developmental programs to cell injury in PD. Future study might set out to further explore the detailed identity of this neuronal population and its implication in PD.

### Inverse Alterations of Interaction Quantity and Strength on a Global Scale in Parkinson’s Disease

As previously mentioned, most scRNA-seq studies of SN mainly focused on the cell-type-specific gene expression patterns in PD. However, recent progress in algorithm has enable the study of intercellular communication (Kumar et al., 2018; Raredon et al., 2019; Wang et al., 2019; Browaeys et al., 2020; Cabello-Aguilar et al., 2020; Efremova et al., 2020; Ren et al., 2020) that are mediated by signaling molecules (ligands) binding to their cognate receptors. The CellChat is one of the latest techniques with some unique advantages over others (Jin et al., 2021). To our knowledge, there has been rare studies characterizing the cell-cell communication with scRNA-seq data in PD research. Because cell–cell interactions often influence the development, differentiation, state transition, and heterogeneity of individual cells, which might be perturbed under pathologic conditions, studying intercellular communication is a novel and meaningful angle of probing the mechanisms of life and disease. We applied the CellChat to our integrated dataset and analyzed the cell–cell communication from a global view to a detailed perspective. On a global scale, we found that the interaction quantity among different cell types is reduced while the interaction strength is enhanced in PD. This novel discovery hinted at the hypothesis that the enhanced strength might constitute a compensatory mechanism for the loss of interaction quantity in PD in an attempt to restore physiological state. Alternatively, excessive signaling intensity might overwhelm the recipient cells, resulting in adverse effects despite a positive intention. This finding might somewhat reshape our understanding of cellular dysfunction in PD.

### Alterations in Intercellular Signaling Networks Involving Dopaminergic Neuron Reveal Mechanisms of Parkinson’s Disease From a Novel Perspective

The global alteration of cell–cell communication is comprised of changes in individual signaling pathways between different cell clusters. The global alteration without distinguishing the cell types cannot reflect the detailed and specific changes mostly relevant to PD, and it is impossible to study all the interactions without any emphasis. Therefore, we placed the primary victims DaN in the center of analysis and focused on interactions involving them. We found that some signaling pathways, including some growth or differentiation factors, neurotrophic factors, neuropeptides and chemokines, are greatly altered in PD compared with those under physiological condition.

Among the growth or differentiation factors, we found that the intercellular activin signals among neurons are generally decreased, whereas those between DaN and non-neuronal cells are generally increased in PD. This phenomenon hinted at two possible scenarios that either the activin signals among neurons transfer to non-neuronal cells as a primary pathologic alteration, or the increased activin signals involving non-neuronal cells constitute a compensatory response to the loss of activin signals among neurons in PD. We also found that the outgoing and incoming activin signals involving DaN are both increased in PD. Activins are secreted ligands of transforming growth factor-β (TGF-β) family members. Activins regulate spine formation, adult neurogenesis, late-phase long-term potentiation and suppress neuroinflammation (Abdipranoto-Cowley et al., 2009) in the brain (reviewed in Ageta and Tsuchida, 2011). Activins also protect DaN and other neurons *in vitro* or in the SN from apoptosis and oxidative toxins like SIN-1, 6-OHDA and MPTP/MPP^+^ (Krieglstein et al., 1995; Kupershmidt et al., 2007; Stayte et al., 2015, 2017). Taken together, activins might play a protective role in DaN loss.

The intercellular signals of another growth factor, the EGF, are greatly altered among neurons but are hardly altered between DaN and non-neuronal cells in PD compared with physiological condition. Physiologically, EGF/EGFR signaling acts on neural stem cells, neural progenitor cells, neurons and glial cells to regulate their differentiation, maturation, growth, survival and functions (reviewed in Romano and Bucci, 2020; Scalabrino, 2020). The levels of EGF and EGFR are decreased in the postmortem brains of PD patients (Iwakura et al., 2005). Abnormalities of several PD-associated proteins alter the endolysosomal trafficking of EGFR and its downstream signaling transduction such as PI3K/Akt pathway, which, together, might underlie the loss of DaN (reviewed in Romano and Bucci, 2020). Given these findings, our analysis with CellChat confirms that the altered EGF/EGFR signals might contribute to DaN loss in the SN.

Another group of signaling molecules NRGs are EGF family related proteins. We found that NRG signals are abundant among astrocytes and OPCs. The actions of NRGs on ODCs were previously reported. NRGs influence the life of ODCs and its progenitor cells, including proliferation, survival, development, and differentiation (Canoll et al., 1996, 1999; Vartanian et al., 1999; Flores et al., 2000). Blockade of ErbB signaling in ODCs not only causes ODCs abnormalities, but also alters dopaminergic function in the brain (Roy et al., 2007). Cortical neurons can release glial growth factor, a subtype of NRG, for pro-oligodendrocytes (Canoll et al., 1996). We also found that DaN showed enhanced NRGs release toward other neurons and non-neuronal cells under PD condition. It was found that NRGs participated in the development and survival of neuronal or non-neuronal cells (reviewed in Buonanno and Fischbach, 2001). NRGs/ErbB signaling pathway is neuroprotective and neurotrophic for midbrain DaN (Zhang et al., 2004; Carlsson et al., 2011; Depboylu et al., 2015), and can regulate dopamine neurotransmitter homeostasis (Ledonne et al., 2015; Skirzewski et al., 2018). However, why DaN send more NRGs to other cells according to our finding is hard to be explained.

Another intercellular signal, Visfatin, is both an intracellular enzyme in NAD^+^ biosynthesis pathway and an extracellularly secreted factor upon stresses, nutritional alteration and inflammation in an autocrine or paracrine manner (reviewed in Carbone et al., 2017). We found that endothelial cells are the only releasing center of Visfatin to other cells in healthy SN, which is hardly altered in PD. Meanwhile, the UnN2 neurons and microglia become additional visfatin releasing centers in PD. In the literature, extracellular Visfatin was shown to act on endothelial cells as a growth factor with proliferative, pro-survival, pro-migrative and proangiogenic effects. In addition, Visfatin was expressed in endothelial cells in the brain (Zhang et al., 2010). But whether endothelial cells are sources of extracellular visfatin and the roles of endothelium-associated visfatin signals in the SN were rarely studied before. Visfatin can confer neuroprotection in ischemic and/or reperfusion stroke with anti-apoptotic (Fan et al., 2011; Erfani et al., 2015) and pro-autophagic actions (Wang et al., 2012). Besides, Visfatin also protects cells via anti-oxidant action (Buldak et al., 2013). However, extracellular Visfatin can also elicit inflammation and promote chemotactic pathway (reviewed in Carbone et al., 2017). The emergence of microglia-associated visfatin signals might imply Visfatin as an inflammatory signal in PD, proof of which requires further investigation.

The neurotrophic GDNFs signal through the ligand-binding subunit GDNF family receptor α1 (GFRα1) and receptor tyrosine kinase RET for signal relay. The GDNF/GFRα1/RET complex leads to downstream signaling of Ras/MAP kinase and PI3K/AKT pathways, and a series of cellular and molecular activities. Current opinions about GDNF hold that it supports and regulates midbrain DaN, and confers neuroprotection against toxic insults in the Parkinsonism animal models. However, whether GDNF is key to the development and maintenance of dopaminergic neurons in midbrain is still unresolved (reviewed in Kramer and Liss, 2015; Ibanez and Andressoo, 2017). GDNF was later found endowed with neurotrophic effects toward other types of neurons as well (Henderson et al., 1994; Arenas et al., 1995; Mount et al., 1995). In our data, GDNF signals among neurons are all abolished in PD. Among the non-neuronal cells, OPCs are the main source of GDNF for DaN under physiological condition, which is not completely agreeable with the literature. It was found that the striatal ODC lineage cells ODC-type 2 astrocytes release soluble neurotrophic factors for midbrain DaN (Takeshima et al., 1994; Sortwell et al., 2000). Later, GDNF was found secreted by ODCs (Wilkins et al., 2003; Ubhi et al., 2010; reviewed in Du and Dreyfus, 2002) and astrocytes (Schaar et al., 1993; Chen et al., 2006; Zhang et al., 2012) but not by OPCs (Wilkins et al., 2003). Both OPCs and differentiated ODCs are able to promote survival of cortical neurons via soluble factors-mediated PI3K/Akt pathway activation, but this neurotrophic effect wasn’t mediated by GDNF. Instead, ODCs-derived GDNF facilitates the axonal integrity and length in neurons (Wilkins et al., 2003). ODCs also secrete other neurotrophic and growth factors to nearby neurons, and are implicated in NDs (reviewed in Du and Dreyfus, 2002; Bankston et al., 2013). Together with our data, the source of GDNF and its effects on neurons need to be further clarified. Our data showed that DaN receive and give out less GDNF signal in PD, indicating that the nigral DaN are deprived of GDNF nurturing.

Chemokines are involved in PD via mechanisms of immunomodulation and neuroinflammation (reviewed in Chamera et al., 2020). In the CNS, CX3CL1 is predominantly expressed and produced by neurons, whereas CX3CR1 is principally expressed on microglia, enabling their response to CX3CL1 (Harrison et al., 1998; Shan et al., 2011). The CX3CL1-CX3CR1 pathway bridges the neuron-microglia dialogue, and is essentially implicated in the regulation of proper activation and functions of microglia (reviewed in Chamera et al., 2020). Our data clearly reflects this expression and crosstalk pattern, and shows that the DaN-to-microglia CX3C signal strongly appears in PD, so does the other direction to a lesser extent. This is in line with the finding in the neurotoxin PD model where MPP^+^ increase the expression of CX3CL1 and CX3CR1 (Shan et al., 2011). CX3C signaling-mediated neuron-microglia communication and its roles in microglia regulation have vital implication for NDs including PD (reviewed in Liu et al., 2019; Luo et al., 2019). However, whether CX3CL1-CX3CR1 pathway play a protective (Pabon et al., 2011; Morganti et al., 2012; Castro-Sanchez et al., 2018) or destructive (Shan et al., 2011; Thome et al., 2015) role in different PD animal models is still controversial (reviewed in Liu et al., 2019; Luo et al., 2019). Further studies may set out to address whether its alteration, either increase or decrease, is a primary pathologic event or secondary compensatory response in PD.

Neuropeptides are short amino acids chains in the nervous systems with neuromodulation abilities (van den Pol, 2012; Hallberg, 2015). Our data showed that the autocrine signal of the neuropeptide CCK is lost in DaN but emerges in ExN in PD. Although there has been few study of intercellular CCK signals in PD, there are abundant evidences of dopamine-CCK interaction (reviewed in Crawley, 1991), which were conducted more than 30 years ago. On the one hand, CCKs influence dopamine metabolism, release, receptor activity and dopaminergic excitation via acting on CCK receptors. CCKs exist in dopaminergic neurons located in the VTA and some region of SN in the midbrain (Hokfelt et al., 1980a,b), which was reflected in our result. In these CCK-positive neurons, sulfated CCK plays a role in promoting the electric activity of these dopaminergic neurons in the midbrain (Skirboll et al., 1981; Hommer et al., 1986). Interestingly, CCK can also potentiate the autoinhibitory effects of apomorphine (Hommer et al., 1986). In terms of dopamine metabolism, CCK-related peptides can decrease dopamine turnover in the midbrain and several other regions, but they increase dopamine turnover in the striatum (Fuxe et al., 1980; Fekete et al., 1981; Kovacs et al., 1981). In terms of dopamine neurotransmission, CCK-8 peptides increase dopamine release in the SN (You et al., 1996) or in the striatum (Kovacs et al., 1981). In addition, CCK-8 can modulate the dopamine binding property of the striatal post-synaptic dopaminergic receptor (Fuxe et al., 1981). At the behavioral level, CCK-related peptides regulate locomotor function: CCK-8 suppresses L-DOPA-induced locomotor activity and antagonizes the potentiating effects thyrotropin on this phenomenon (Itoh and Katsuura, 1981). Non-sulfated CCK-8 can facilitate apomorphine-induced stereotyped behavior (Kovacs et al., 1981). In addition to locomotor activity, CCK-related peptides also have antipsychotic effects when administered to nucleus accumbens of rats (Van Ree et al., 1983).

On the other hand, dopamine in turn also regulates CCK: dopamine promotes CCK production tonically via acting on D2 dopaminergic receptor in the striatum (Ding and Mocchetti, 1992) and CCK release (Klaff et al., 1982). Besides the aforementioned dopamine-CCK duo, there exists a dopamine-CCK- acetylcholine (ACh) trio in the striatum. CCK-8 enhances striatal ACh release upon electric stimulation via both CCK_A_ and CCK_B_ receptor, an effect under the inhibitory regulation of dopaminergic tonic activity in the striatum (Petkova-Kirova et al., 2012). This study showed that CCK-8 was involved in the ACh-DA imbalance in PD, and CCK antagonist might be an alternative therapeutic option in regulating ACh-DA imbalance.

Besides complex dopamine-CCK interaction, several lines of other evidences also implicate CCK in PD. Neuropathologic study showed that CCK-8 was depleted selectively in the SN, in line with our findings, but not in other mesencephalic and forebrain areas, including striatum and nucleus accumbens (Studler et al., 1982; Fernandez et al., 1992). This might be consequent to dopaminergic neuron loss. In the periphery, the CSF CCK level is also significantly reduced in PD patients (Lotstra et al., 1985). Epidemiological study showed that the polymorphism at the –45 locus of *CCK* promoter is significantly different not only between PD and healthy controls, but also between treated PD patients with hallucination and those without in Japanese population (Fujii et al., 1999). The *TT*/*TC* genotype at this locus is associated with an increased risk for visual hallucination in PD patients in Chinese population; moreover, the combination of this genotype with *TC*/*CC* genotype at the 779 locus of *CCKAR* also indicates a higher risk of developing hallucination in PD in Chinese population (Wang et al., 2003). However, such differential distribution and association were not found in white population (Goldman et al., 2004, 2011). Laboratory data showed that 6-OHDA treatment alone or together with L-DOPA treatment significantly increased the level of CCK or CCK-derived peptides in the SN (Taylor et al., 1992; Nilsson et al., 2009; Yu et al., 2020). Besides observatory study, some mechanism study might help understand the reasons behind the alteration of CCKs. DJ-1 (encoded by *PARK7*) activated *CCK* expression by binding to Ras-responsive element (RRE) binding protein 1 (RREBP1), a transcription factor that stimulated *CCK* transcription when associating with the RRE located on the *CCK* promoter (Yamane et al., 2013). Since loss of DJ-1’s function has been widely observed in sporadic and familial PD (reviewed in Huang and Chen, 2021), this mechanism might help partially explain the loss of CCK in dopaminergic neurons in PD. Besides, recent bioinformatic study also found CCK as one of the hub genes of PD with the potential to be explored as a druggable target for PD (Odumpatta and Arumugam, 2021). Despite so many evidence implicating CCK in PD, our bioinformatic discovery that the CCK signaling transferred from dopaminergic neurons to excitatory neurons is still an unreported novel finding worthy of exploratory study.

### Limitation and Future Direction

Although we have gathered as much sc/snRNA-seq data of human SN as possible, used the latest and sophisticated methods and algorithms, and portrayed both the cell composition and cell–cell communication under physiological and PD condition, our analysis was not without limitations. First, because of limited time and energy, we only selected some highly relevant intercellular signaling pathways with the most intense alteration for detailed characterization. These pathways shown by our analysis, as a tip of the iceberg, mirrored a much broader range of intercellular messengers that were distorted under PD condition. There were likely other novel but yet uncharacterized signaling pathways in our data that were highly PD-relevant and greatly altered. Future analysis may set out to dig deeper into our data, mine for more disease-associated intercellular signaling pathways, and uncover more alterations in cell–cell communication within SN. Such studies will greatly boost our knowledge of cell–cell relationship under PD condition, which are very likely to translate into therapeutic potential.

A Second limitation in this study lies in the limited data size. Although we have already expanded the sample size through integrating three published datasets, the numbers of single cells analyzed in our study were still very small. This was particular true for TH-positive DaNs. The reason might be that the proportion of DaN in all the cells in SN was indeed small. Alternatively, the authors who provided the GSE157783 dataset (which was the only dataset containing PD samples) (Smajić et al., 2020) found that the nucleus of DaNs were lost in the 15 μm-thick sectioning procedure, as discussed earlier. In addition, the loss of DaN in PD further contributed to the limited number of DaNs. Besides, the source of PD samples was limited, with only one dataset (GSE157783) containing samples from five idiopathic PD patients. Most sc/snRNA-seq studies of PD focus on probing the cell type-specific contribution to the genetic risk of PD with tissues from normal murine or healthy donor, and very few studies were performed on tissues from parkinsonism murine models (Zhong et al., 2021) or postmortem PD patients (Smajić et al., 2020). In terms of these, future studies might try to harvest more SN samples from parkinsonism animal models or postmortem human brains to characterize more PD-related pathogeneses at single-cell resolution.

Third, the CellChat algorithm infers cell-cell communication with a manually curated database containing ligand-receptor interaction information and the co-differential expression of ligand and receptor genes. The results of CellChat inference, despite its robustness shown by its creators (Jin et al., 2021), still require previous literature support or further experimental validation. Therefore, future studies might set out to confirm and interpret the results in our data with laboratory evidences, and endow these data with experimental and translational significance.

In spite of these limitations, many key results yielded in our study strongly supported the robustness and reliability of our bioinformatics results. For example, our CellChat data showed a bidirectional CX3C communication between DaNs and microglia, which was in line with the established neuron-microglia dialogue via CX3CL1-CX3CR1 signaling between these two types of cells (reviewed in Chamera et al., 2020). Besides, our data showed that DaNs were deprived of GDNF nurturing in PD, echoing with the neuroprotective roles of GDNF (reviewed in Kramer and Liss, 2015; Ibanez and Andressoo, 2017). Other intercellular signaling pathways, such as activins, EGF, NRG, etc. all had already been implicated in PD albeit to different extent as discussed in detail earlier. Therefore, the intercellular communication revealed by CellChat analysis had mostly been associated with PD or neurodegeneration before. Other evidence also strengthened the robustness of our data: for example, the cell identities we annotated in our integrated dataset were highly aligned with the original datasets. In short, multiple aspects of our data were fully aligned with previous laboratory evidences, highly supporting the authenticity of our analysis to reflect the *bona fide* biological or pathologic phenomenon. In this way, our results also constitute a faithful application of the Harmony and the CellChat algorithms, in favor of their robustness. All in all, despite the presence of some limitations, our integrated sc/scRNA-seq and cell–cell communication data are convincing, paving the way for future detailed and deeper mechanistic studies.

Although we have extensively studied the cell–cell communication via signaling molecules under PD condition, there was another aspect of intercellular pathogenesis concerning the cell–cell propagation of pathologic forms of α-synuclein left untouched in our previous analysis. α-synuclein transmission among cells is one of the key mechanisms underlying the progression of PD. Considering its importance in the field of PD research, we wonder whether the thriving sc/snRNA-seq technology can offer some novel and distinct view to at least some of its mechanisms.

α-Synuclein propagation among cells is a complex pathologic process where multiple biological events are engaged. It contains sequential steps including the release of pathologic α-synuclein from cells and its uptake by receptor cells. On each step there exists multiple parallel mechanisms: α-synuclein fibrils can travel between cells via exosome release and uptake, via tunneling nanotube, via exocytosis and different forms of endocytosis, via leakage from damaged cells, via direct membranous translocation, and via synaptic contact (reviewed in Visanji et al., 2013; Uemura et al., 2020). These processes involve a variety of molecular events, such as posttranslational modification and conformational alteration of α-synuclein, protein-protein interaction, and interaction with membrane, etc. SnRNA-seq alone studies biological events mainly via characterizing transcriptional activities. Though α-synuclein propagation is very likely to involve transcriptional alteration of a certain genes, which is a pioneering field awaiting exploration, simple alterations in gene expression might not fully account for the multi-stage and complex mechanisms of α-synuclein propagation.

Among the complex mechanisms of α-syn propagation, an emerging topic concerns whether there are some selective receptors that involve α-syn uptake (reviewed in Hijaz and Volpicelli-Daley, 2020). Studies in the last decades told us it is the case. For example, α-syn fibrils bind to heparan sulfate proteoglycans (HSPGs), which facilitates α-syn uptake and propagation (Holmes et al., 2013). Lymphocyte-activation gene 3 (LAG3), neurexin 1β and amyloid beta precursor-like protein 1 (APLP1) were also identified as binding partners for α-synuclein PFF (Mao et al., 2016). Among them, LAG3, which is the most selective receptor for α-synuclein preformed fibrils (PFF), can mediate endocytic uptake of α-synuclein PFF and therefore its transmission, toxicity, and associated PD phenotypes (Mao et al., 2016). In addition, N-terminally acetylated α-synuclein recognizes and binds to the N-linked glycans at the extracellular glycosylation site of neurexin 1β (encoded by *NRXN1*). This binding, which requires the presence of glycosylation, mediates the internalization of acetylated α-synuclein (Birol et al., 2019). Sparked by the concept and strategy of CellChat to study cell–cell interaction, we wondered if the upregulation of these “receptor” genes participated in α-synuclein transmission. Correspondingly, we hypothesized that *LAG3* and *NRXN1* were upregulated in PD samples compared with healthy samples, and we profiled their expression. However, we did not find any difference in these two genes’ expression between healthy and PD samples (data not shown). It might be that LAG3 or neurexin 1β-mediated α-syn propagation only account for a very small portion of α-syn transmission. Alternatively, the roles of LAG3 or neurexin 1β in α-syn propagation have little to do with their transcriptional alterations. In fact, it was the posttranslational modification of neurexin 1β, i.e., glycosylation, that accounts for its α-syn internalization activity (Birol et al., 2019). Therefore, transcriptional alteration alone might not fully account for the transmission of pathologic α-synuclein, and multi-modality single cell technology might be necessary to offer novel insights into its mechanisms.

## Conclusion

Our bioinformatics analysis highlighted the implication of altered cell-cell communication in PD pathogeneses, showing that DaNs were not alone, and other cells were not mere onlookers or bystanders along the way down to degeneration. It filled the blank space and provided the preliminary evidence of intercellular pathogeneses in the story of PD. It would also help researchers see beyond DaNs themselves and consider more about the complex relationship between DaNs and other cells in PD. Furthermore, our data hinted at the potential that modulating cell–cell communication might constitute a supplementary therapeutic strategy to the current existing disease-modifying strategy under trial, which, together, might increase the possibility of effective disease modification in PD.

## Materials and Methods

### Quality Control and Data Correction

Quality control and data correction for single-cell samples were based on the number of detected genes, the number of detected molecules, and the percentage of mitochondrial, ribosomal and hemoglobin genes from each single-cell sample. Individualized filtering plans were applied to different datasets based on their unique features (Supplementary Figure 1). In detail, for dataset GSE116470, samples with less than 1000 genes, more than 3000 gene, less than 200 molecules, more than 1% mitochondria genes and more than 2% ribosomal genes were removed. For dataset GSE140231, samples with less than 600 genes, more than 2000 genes, less than 200 molecules, more than 0.5% mitochondrial genes and more than 2% genes were removed. For dataset GSE157783, single-cell samples with less than 1000 genes, more than 6000 genes, less than 200 molecules, more than 0.05% ribosomal genes were removed. The remaining data in the three datasets were later used to produce a combined dataset.

### Normalization, Integration, Dimensionality Reduction, Clustering and Visualization, and Cluster Annotation

After quality control, Seurat R package v4.0.2 was used to process the data. Sctransform (Hafemeister and Satija, 2019), which enables the recovery of sharper biological distinction, was used for normalization. Then, Harmony, an integration algorithm, was used to integrate the above-mentioned three datasets and perform the dimensionality reduction (Korsunsky et al., 2019; Supplementary Figure 2). Clustering and visualization of the integrated dataset were realized by Uniform Approximation and Projection method (UMAP) at a resolution of 0.05 and a dimension of 20. Canonical marker genes for neuronal and non-neuronal cells were used to annotate each cell cluster.

### Differential Gene Testing and Gene Ontology/Kyoto Encyclopedia of Genes and Genomes Enrichment Analysis

Differential gene testing was performed through the FindMarkers() function in the Seurat. The FindMarkers() function uses the non-parametric Wilcoxon rank sum test as a default method. The adjusted *p*-values (p_val_adj) it provides were based on Bonferroni correction using all features in the dataset.

Then, GO and KEGG enrichment analysis were conducted to summarize the functions of differential genes in each cluster. The significance level of *P* < 0.05 was taken as the cut-off standard.

### Subclustering of the Neuron Cluster

The procedure of subclustering the neuron cluster (NEU) was the same as that of the pan-cell dataset.

### Analysis of Cell–Cell Communications That Involve Dopaminergic Neuron Mediated by Intercellular Signaling Pathways

To characterize cell–cell communications specifically involving DaN via signaling pathways, non-DaN were removed in the NEU cluster, with the remaining cells comprising DaNs. Then CellChat 1.0.0, an algorithm for analyzing cell–cell communication at single-cell level, was used to infer and quantify cell–cell communication involving DaN. The global intercellular communication network under physiological and PD conditions were quantified, visualized and compared. The information flows for each signaling pathway, which defined as all communication probability among all the pairs of cell groups in inferred network, were calculated and compared between healthy control and PD patients. Euclidean distance between any pair of the shared signaling pathways was computed. Finally, to explore the changes of specific signaling pathways underlying the global alteration, cell–cell communications among neurons, and between DaN and non-neuronal cells, were analyzed and compared respectively, and those greatly altered in PD were picked up for detailed analysis and literature review.

## Data Availability Statement

Publicly available datasets were analyzed in this study. This data can be found here: https://www.ncbi.nlm.nih.gov/geo/query/acc.cgi?acc=GSE126836, https://www.ncbi.nlm.nih.gov/geo/query/acc.cgi?acc=GSE140231, and https://www.ncbi.nlm.nih.gov/geo/query/acc.cgi?acc=GSE157783.

## Author Contributions

SC, LX, and YT conceived and designed the study. LX performed the integration of the three sc/snRNA-seq datasets of human SN published online and further processing and analysis of the integrated sc/snRNA-seq dataset. MH analyzed and interpreted the data, and composed the manuscript. SC and YT revised the manuscript. JL and PH assisted in data analysis. All the authors agreed and approved the final manuscript.

## Conflict of Interest

The authors declare that the research was conducted in the absence of any commercial or financial relationships that could be construed as a potential conflict of interest.

## Publisher’s Note

All claims expressed in this article are solely those of the authors and do not necessarily represent those of their affiliated organizations, or those of the publisher, the editors and the reviewers. Any product that may be evaluated in this article, or claim that may be made by its manufacturer, is not guaranteed or endorsed by the publisher.
