# Supplementary material for: Cell–Cell Communication Alterations via Intercellular Signaling Pathways in Substantia Nigra of Parkinson’s Disease
Source: Front Aging Neurosci. 2022 Feb 25;14:828457. doi: 10.3389/fnagi.2022.828457 (PMC8914319; doi:10.3389/fnagi.2022.828457)
Supplement: Supplementary file 1 [file Data_Sheet_1.docx]

**Supplementary materials**

**Supplementary Figures**


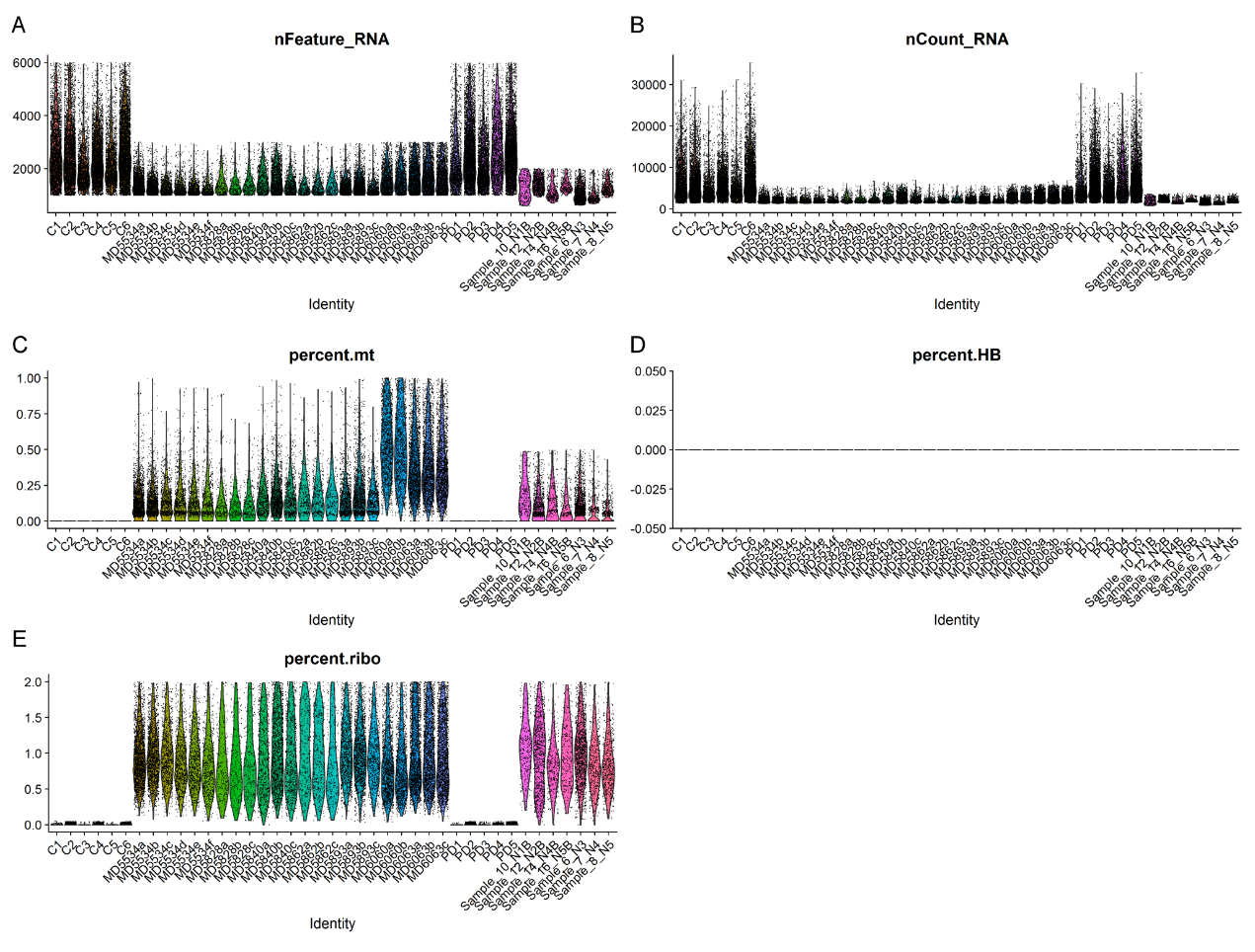


**Supplementary Figure 1. Quality control and data correction.** The quality control of data from three online published single-cell/single-nucleus transcriptomic sequencing (sc/snRNA-seq) datasets were based on the number of genes (nFeature_RNA; A), the number of molecules (nCount_RNA; B), the percentage of mitochondrial genes (percent.mt; C), the percentage of hemoglobin genes (percent.HB; D) and the percentage of ribosomal genes (percent.ribo; E).


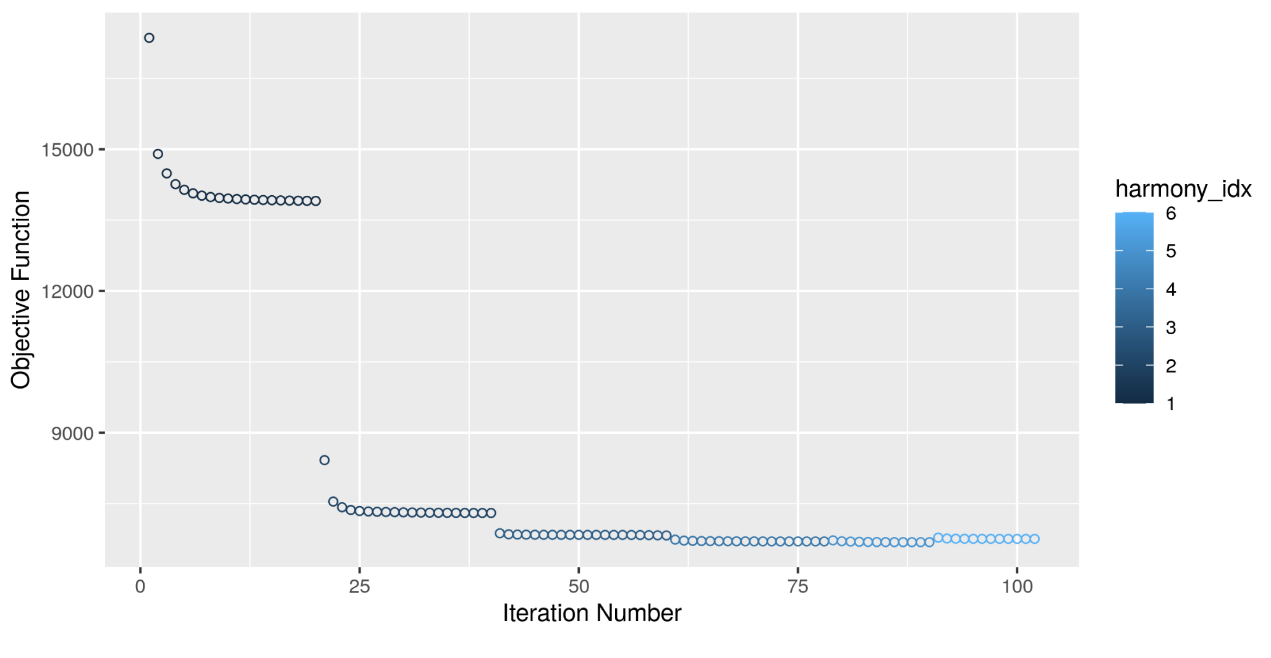


**Supplementary Figure 2. Integration process by Harmony.** The convergence of Harmony objective function turns better with the increase of iteration number. Each point represents a single cluster step, and different colors represent different Harmony iterations.


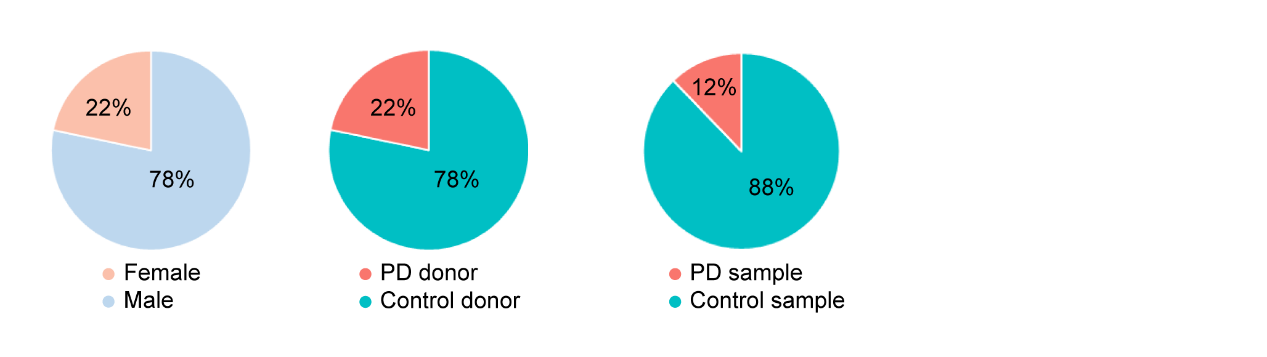


**Supplementary Figure 3.** **Basic information about the gender and source composition in our integrated dataset.**


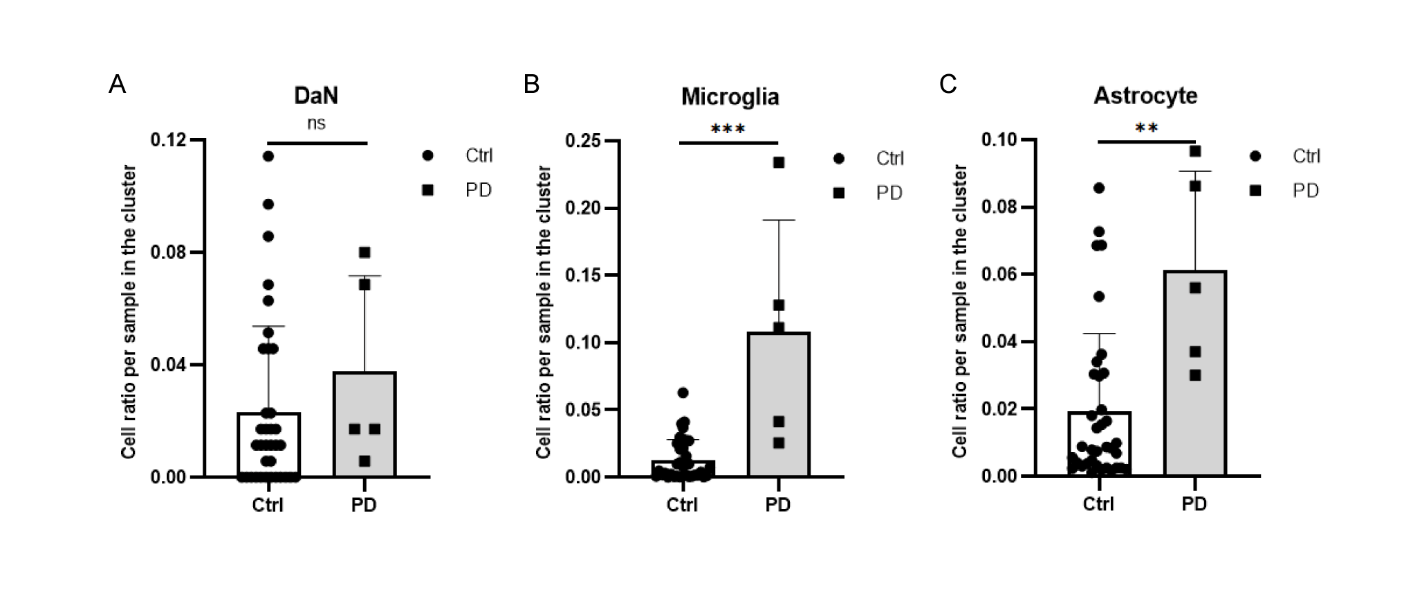


**Supplementary Figure 4**. **Comparison of dopaminergic neuron, microglia, and astrocyte per sample between control (Ctrl) and PD sample.** Mann-Whitney test, p=0.1796 for DaN, p=0.0002 for microglia, p=0.0020 for astrocyte; * p < 0.05, ** p < 0.05, *** p < 0.005; DaN, dopaminergic neuron.


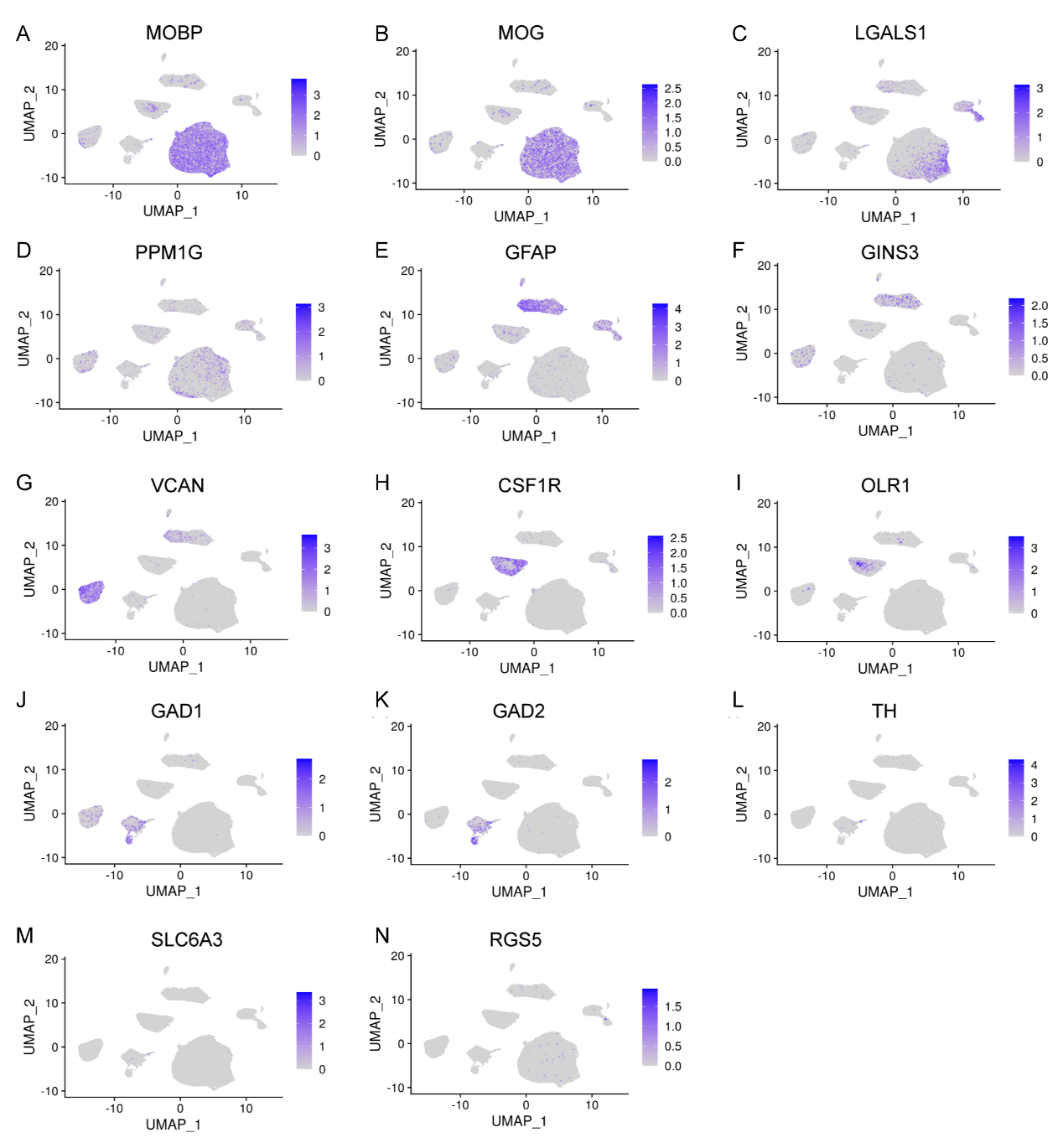


**Supplementary Figure 5. Cell cluster annotation by canonical marker genes for neuronal and non-neuronal cells.** UMAP feature plots showing the expression of ODC markers (*MOBP, MOG, LGALS1, PPM1G*), astrocyte markers (*GFAP*, *GINS3*), OPC markers (*VCAN*), microglia markers (*CSF1R*, *OLR1*), inhibitory neuron markers (*GAD1*, *GAD2*), dopaminergic neuron markers (*TH*, *SLC6A3*), and endothelial markers (*RGS5*) in all clusters.


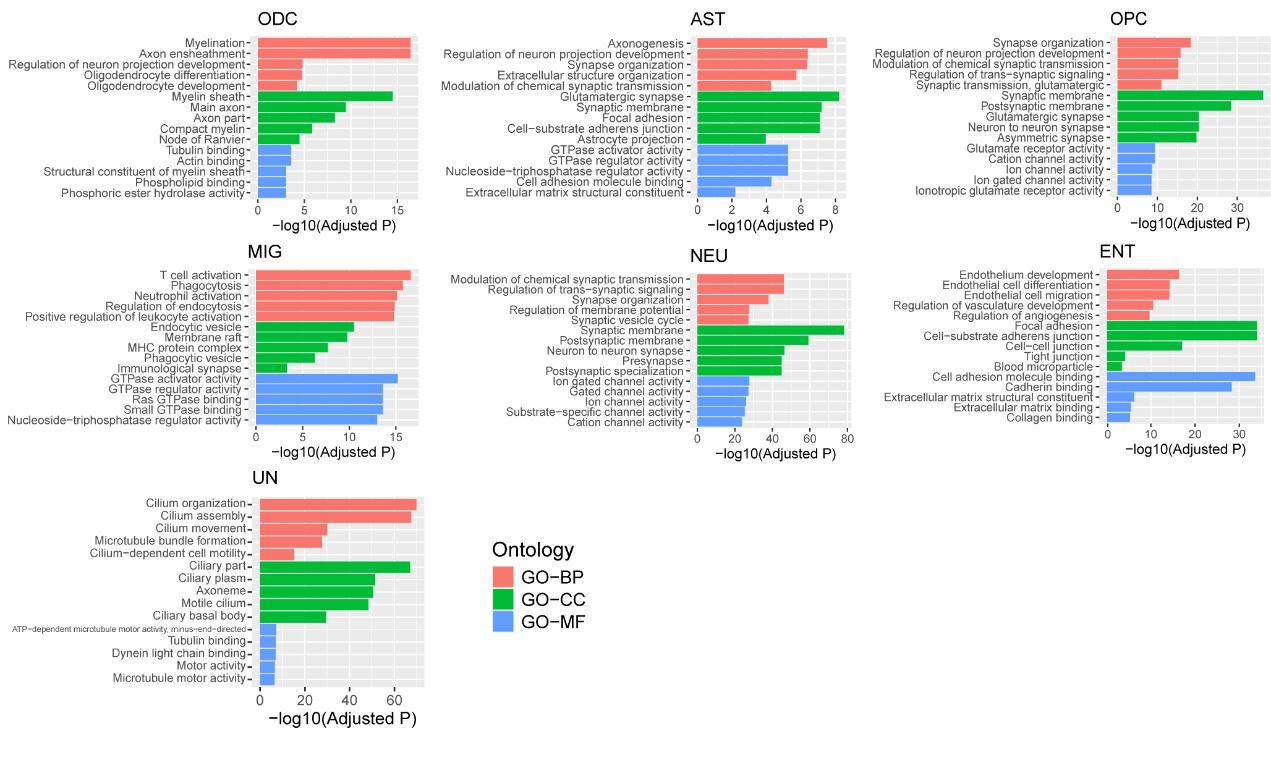


**Supplementary Figure 6. Gene ontology (GO) enrichment analysis of differentially expressed genes in each cell cluster.** (ODC, oligodendrocyte cluster; AST, astrocyte cluster; OPC, oligodendrocyte precursor cell cluster; MIG, microglia cluster; NEU, neuron cluster; ENT, endothelial cell cluster; UN, unidentified cell cluster; BP, biological process; CC, cellular component; MF, molecular function)


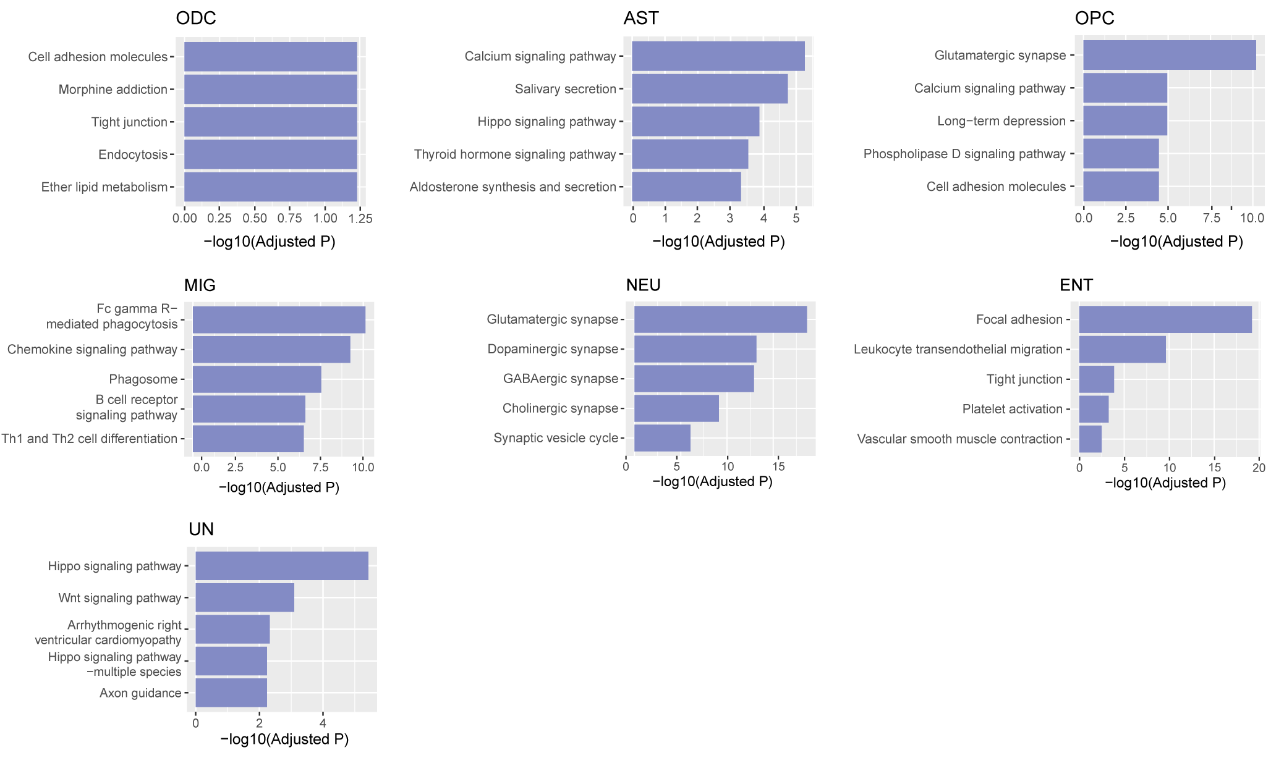


**Supplementary Figure 7. Kyoto Encyclopedia of Genes and Genomes (KEGG) enrichment analysis of differentially expressed genes in each cell cluster.**


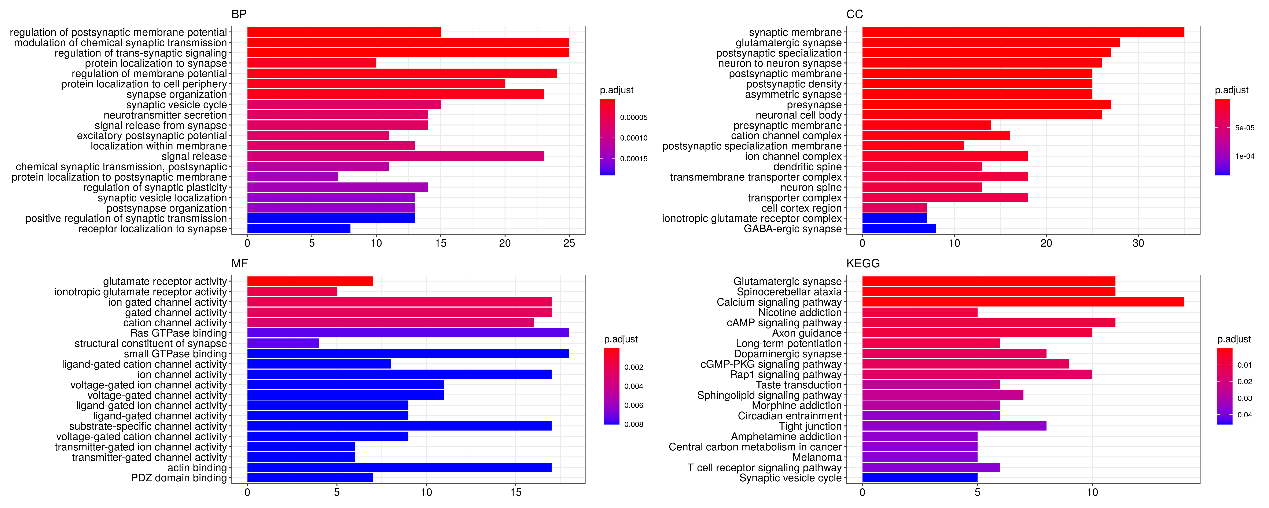


**Supplementary Figure 8. GO and KEGG enrichment analysis of differentially expressed genes in the neuronal subcluster UnN3.**


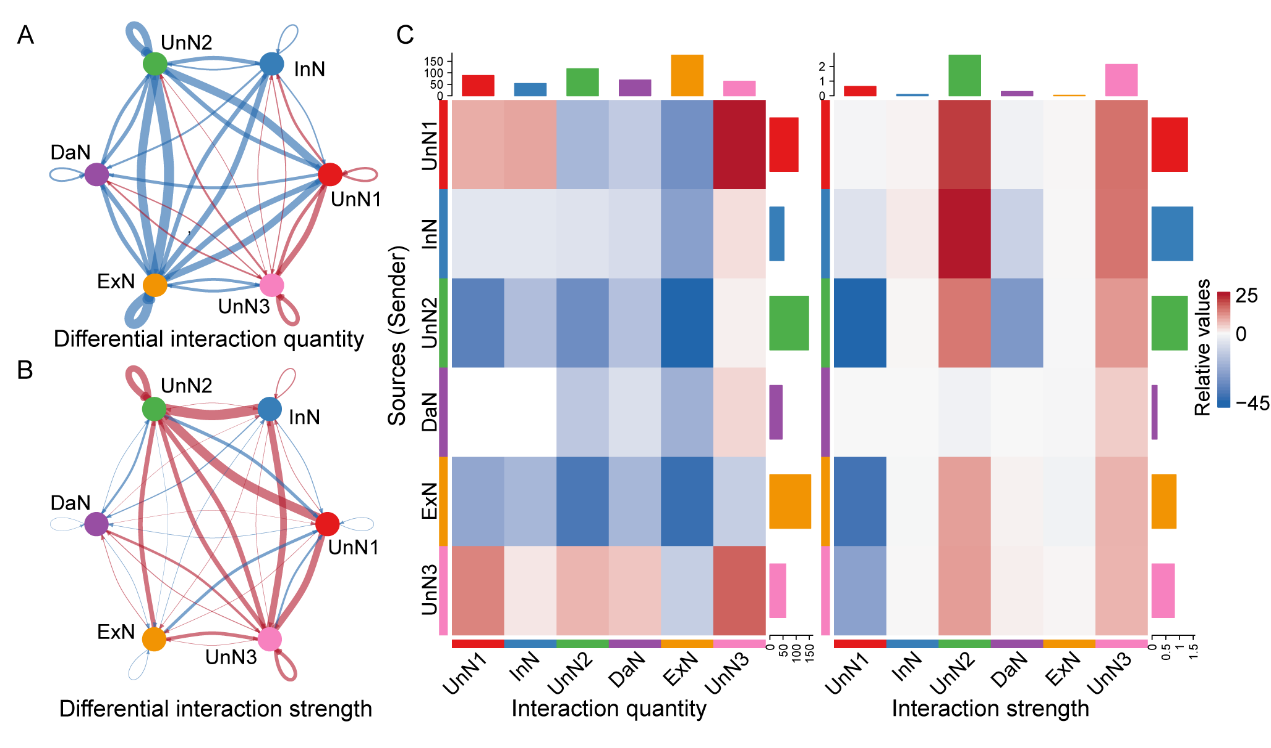


**Supplementary Figure 9. Inference of cell-cell communications by CellChat shows global alterations in signaling pathways-mediated interaction among different neuronal populations in PD.** Circle plots of the interaction quantity **(A)** and interaction strength **(B)** among different neurons. Blue lines indicate that the displayed communication is decreased in PD while whereas red lines indicate that the displayed communication is increased in PD compared with control. **(C)** Heatmaps of the interaction quantity (left panel) and interaction strength (right panel) among different neuronal populations in the SN. Blue color indicates that the displayed communication is decreased in PD while red color indicates that the displayed communications increased in PD compared with healthy control. (InN: inhibitory neuron cluster; DaN: dopaminergic neuron cluster; ExN: excitatory neuron cluster; UnN1, unidentified neuron cluster 1; UnN2, unidentified neuron cluster 2; UnN3, unidentified neuron cluster 3)


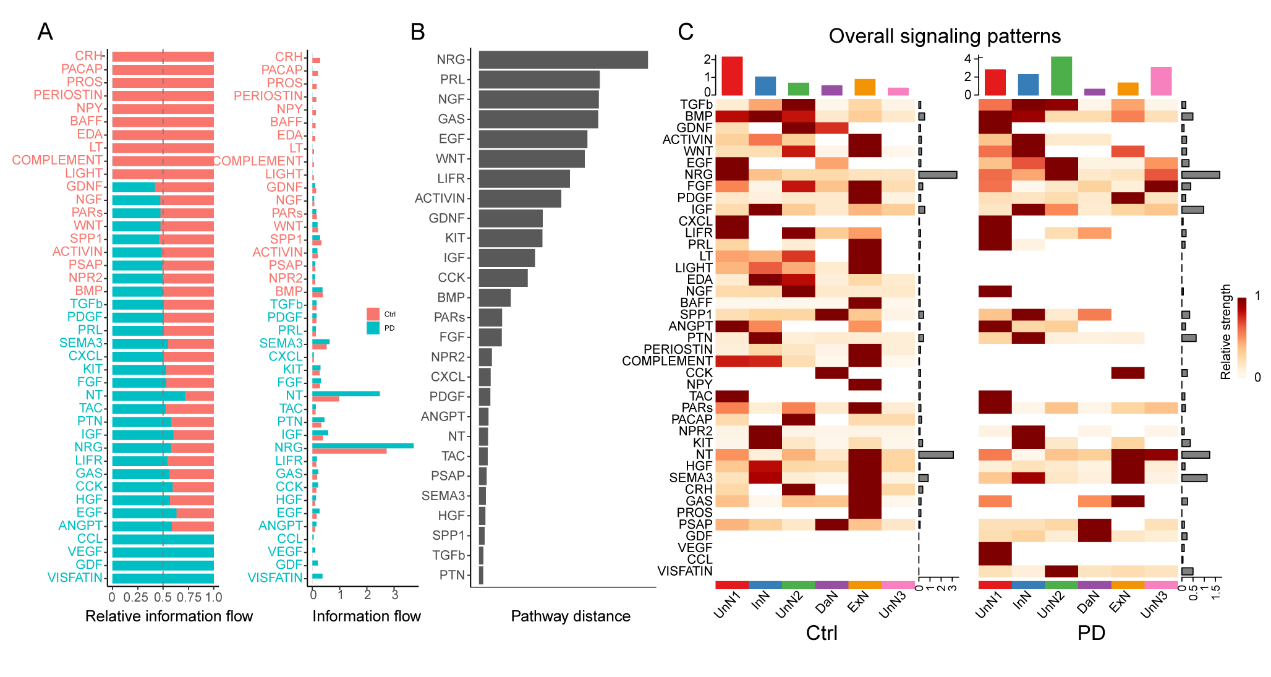


**Supplementary Figure 10.** **Cell-cell communications mediated by individual signaling pathways are altered in PD among different neuronal populations. (A)** Bar plots of the ranking of signaling pathways by overall information flow differences in the interaction networks among different neurons between control (Ctrl) and PD. The top signaling pathways with red-colored labels are more enriched in control sample, the middle ones with black-colored labels are equally enriched in control and PD sample, and the bottom ones with green-colored labels are more enriched in PD. **(B)** Bar plot of the ranking of signaling pathways among different neurons between control and PD by pairwise Euclidean distance. **(C)** Heatmaps of the overall (comprising both outgoing and incoming) signaling flows of each cell population mediated by individual signaling pathways among different neurons in control and PD sample.


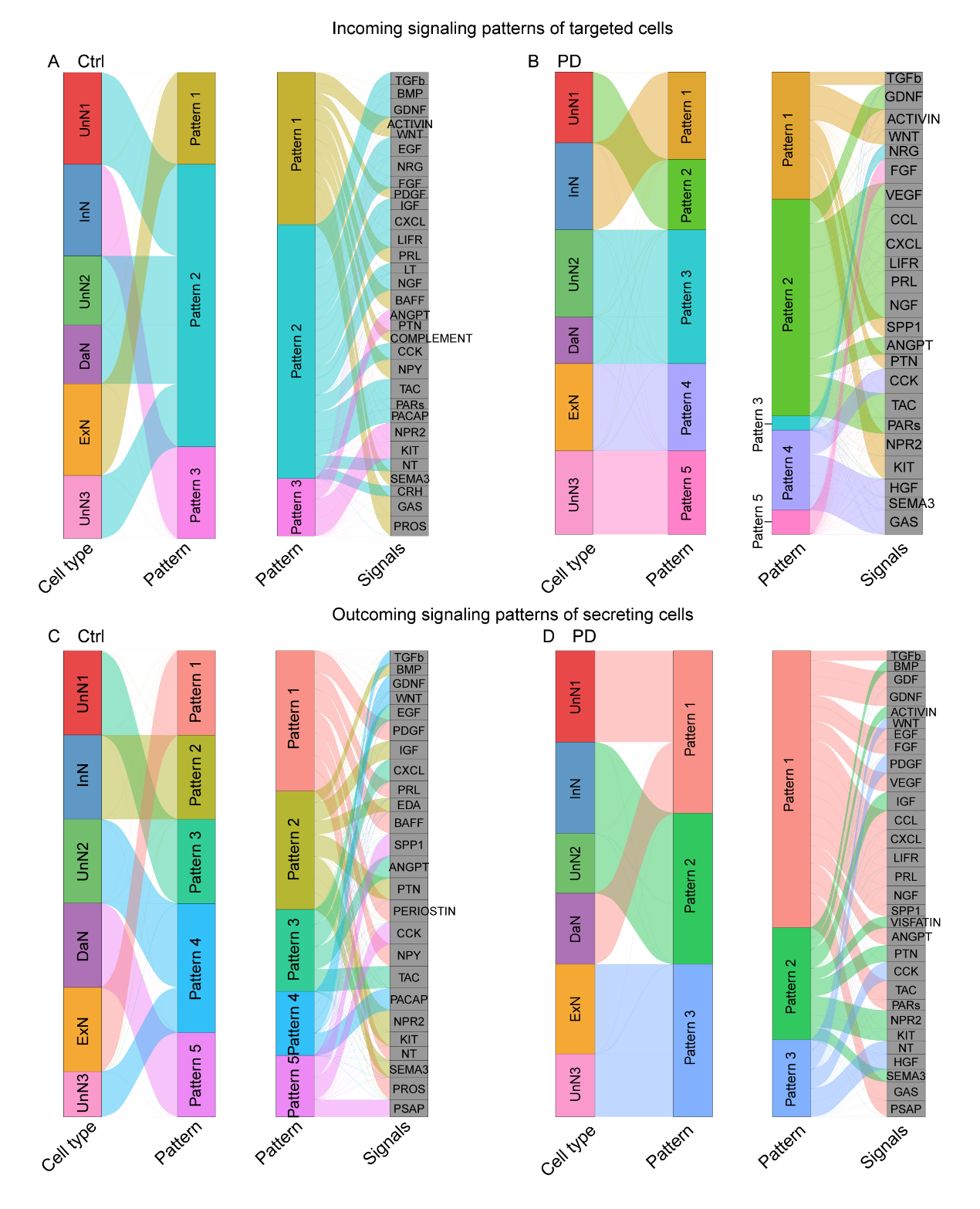


**Supplementary Figure 11. Cell-cell communication patterns among different neuronal populations are altered in PD.** River plots of the inferred incoming communication patterns of the targeted cells among neurons in control (Ctrl) **(A)** and PD **(B)**. River plots of the inferred outgoing communication patterns of the secreting cells among neurons in control **(C)** and PD **(D)** sample. The flow thickness indicates the contribution of the cell group or signaling pathway to each latent pattern.


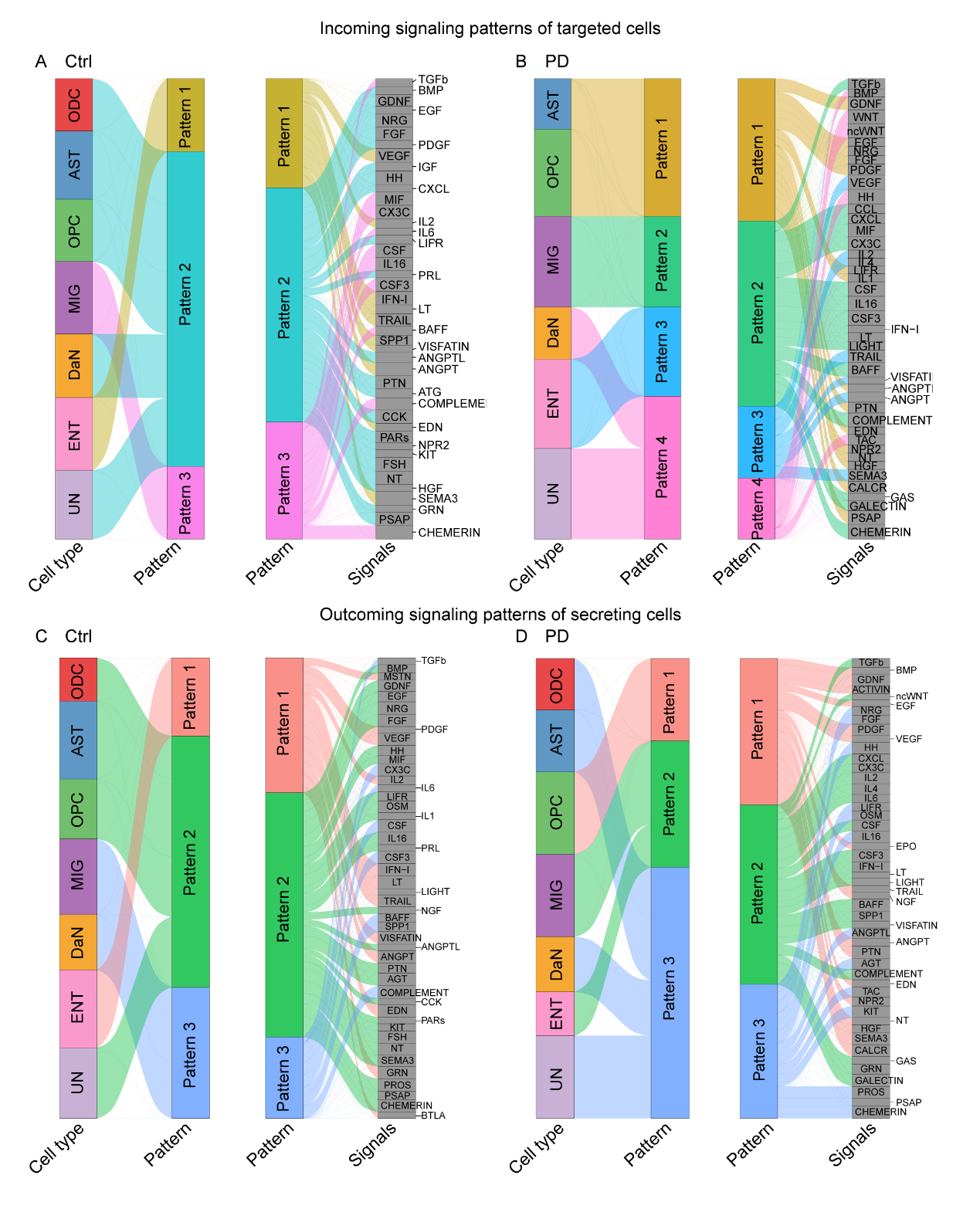


**Supplementary Figure 12. Cell-cell communication patterns between DaN and non-neuronal cells are altered in PD.** River plots of the inferred incoming communication patterns of the targeted cells between DaN and non-neuronal cells in control (Ctrl) **(A)** and PD **(B)** sample. River plots of the inferred outgoing communication patterns of the secreting cells between DaN and non-neuronal cells in control **(C)** and PD **(D)** sample. The flow thickness indicates the contribution of the cell group or signaling pathway to each latent pattern.


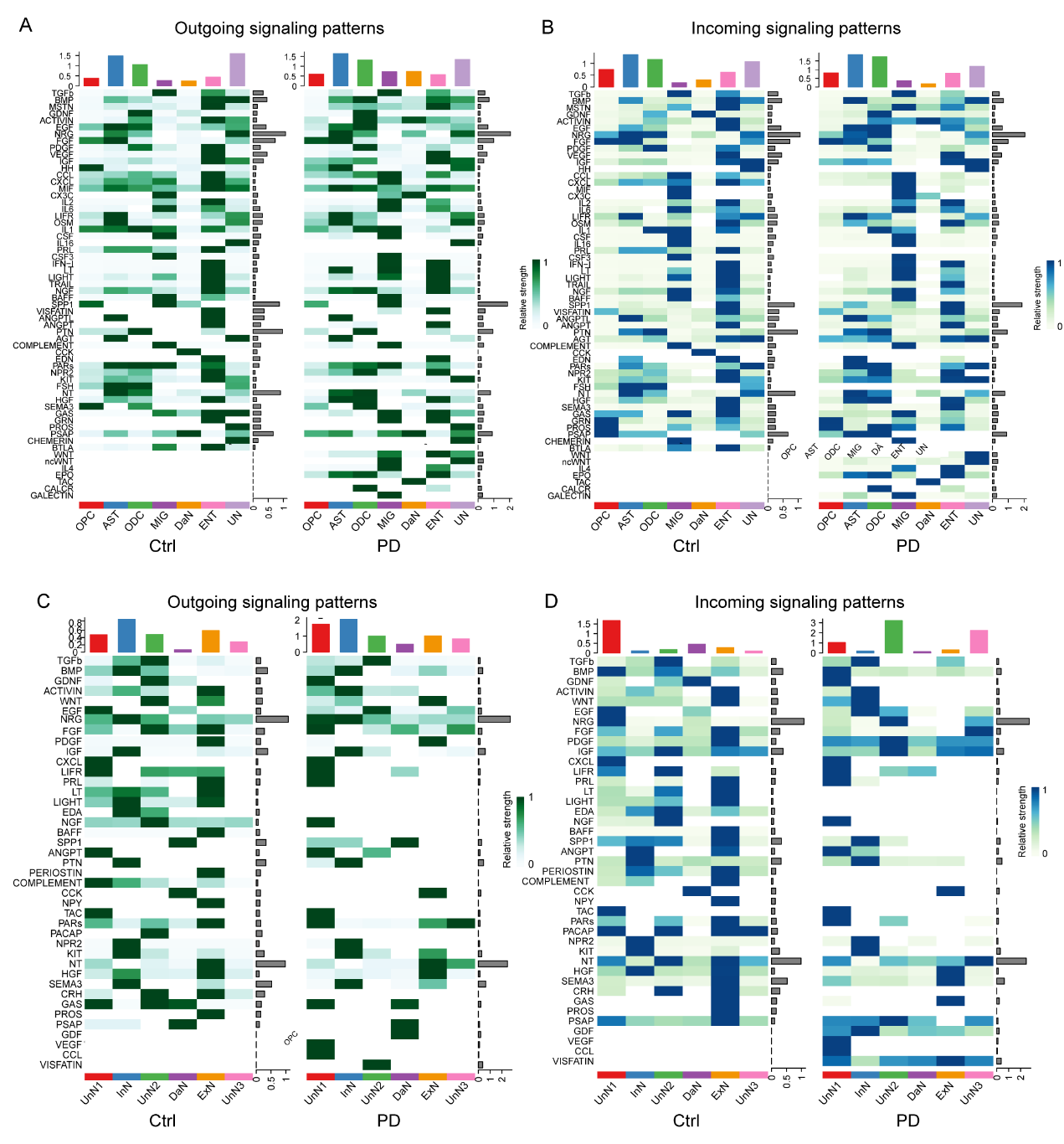


**Supplementary Figure 13.** **The outgoing and incoming intercellular signals of each cluster are altered in PD.** **(A)** Heatmaps of the outgoing intercellular signals from each cluster between DaN and non-neuronal cells in control (Ctrl, left panel) and PD (right panel) sample. **(B)** Heatmaps of the incoming intercellular signals to each cluster between DaN and non-neuronal cells in control (left panel) and PD (right panel) sample. **(C)** Heatmaps of the outgoing intercellular signals from each cluster among different neuronal populations in control (left panel) and PD (right panel) sample. **(D)** Heatmaps of the incoming intercellular signals to each cluster among different neuronal populations in control (left panel) and PD (right panel) sample.


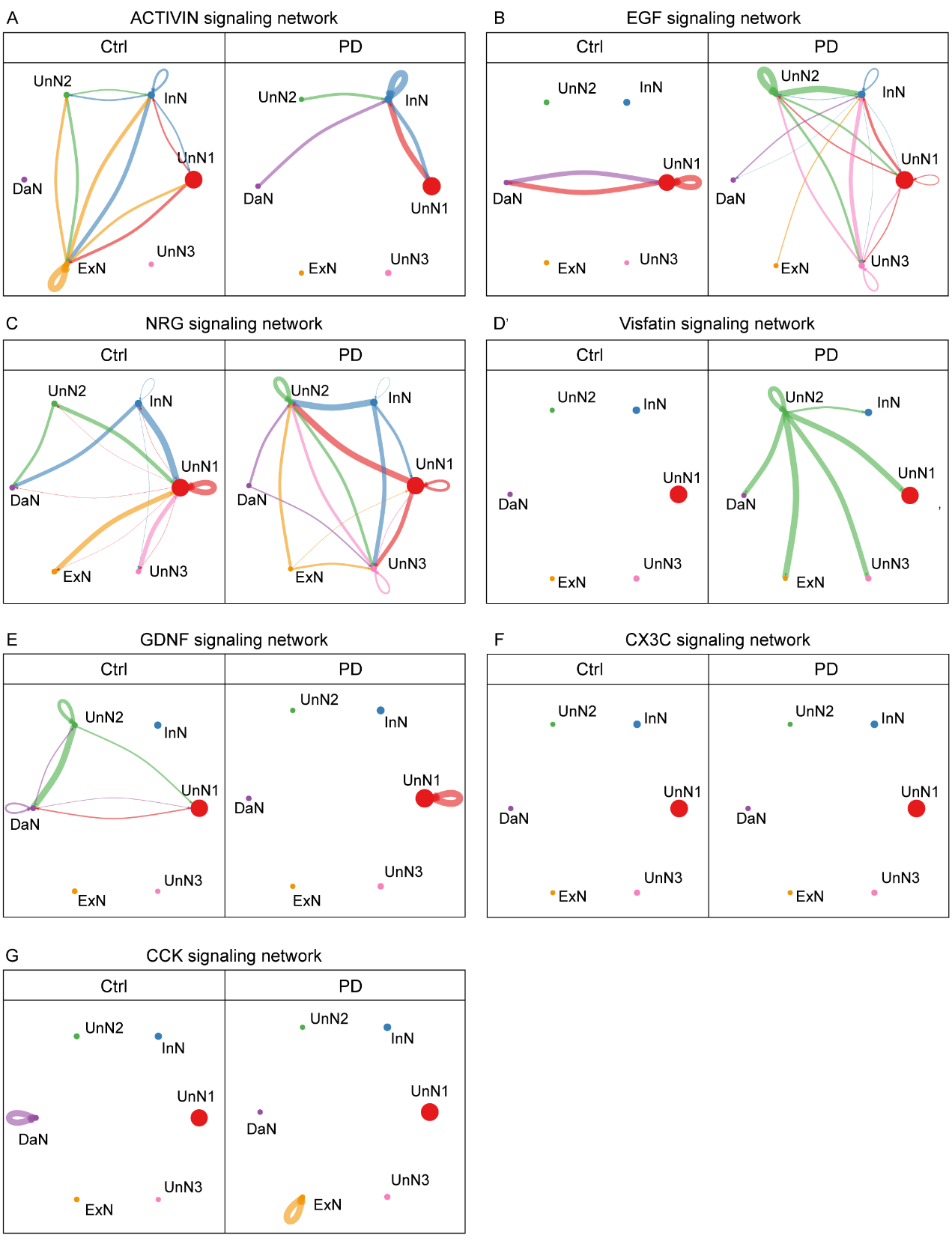


**Supplementary Figure 14. Some cell-cell communication among different neuronal populations mediated by signaling pathways are greatly altered in PD.** Circle plots showing and comparing cell-cell communication alterations among different neuronal populations in the SN mediated by some of the signaling pathways, including activins **(A)**, epithelial growth factor (EGF) **(B)**, neuregulins (NRGs) **(C)**, Visfatin **(D)**, glial cell line-derived neurotrophic factors (GDNF) **(E)**, CX3C **(F)**, and cholecystokinin (CCK) **(G)**.

**Supplementary tables**

**Supplementary Table 1. Basic detailed information for the 3 original snRNA-seq datasets GSE157783, GSE140231 and GSE126836**

**Supplementary Table 2. Basic summary information for the 3 original snRNA-seq datasets (GSE157783, GSE140231 and GSE126836) and integrated dataset**

**Supplementary Table 3. Differential gene testing in each cluster against all other cell cluster in the SN.** Differential gene testing was performed with the non-parametric Wilcoxon rank sum test as a default method. p_val in the first column means unadjusted p value. avg_log2FC in the second column indicates log fold-change of the average expression between the selected cluster and all other cells. Positive values indicate that the gene is more highly expressed in the selected cluster against all other cells. pct.1 and pct.2 in the third and fourth column indicate the percentage of cells where this high expression pattern of a certain gene is detected in the selected cluster and in all other cells, respectively. p_val_adj in the fifth column indicates adjusted p-value, based on Bonferroni correction using all features in the dataset.

**Supplementary Table 4.** **Differential gene testing in each neuronal subcluster against all other neuron subclusters in the SN.** Differential gene testing was performed with the non-parametric Wilcoxon rank sum test as a default method. p_val in the first column means unadjusted p value. avg_log2FC in the second column indicates log fold-change of the average expression between the selected cluster and all other cells. Positive values indicate that the gene is more highly expressed in the selected cluster against all other cells. pct.1 and pct.2 in the third and fourth column indicate the percentage of cells where this high expression pattern of a certain gene is detected in the selected cluster and in all other cells, respectively. p_val_adj in the fifth column indicates adjusted p-value, based on Bonferroni correction using all features in the dataset.
